# Characterizing Intercellular Communication of Pan-Cancer Reveals SPP1+ Tumor-Associated Macrophage Expanded in Hypoxia and Promoting Cancer Malignancy Through Single-Cell RNA-Seq Data

**DOI:** 10.3389/fcell.2021.749210

**Published:** 2021-10-05

**Authors:** Jinfen Wei, Zixi Chen, Meiling Hu, Ziqing He, Dawei Jiang, Jie Long, Hongli Du

**Affiliations:** ^1^School of Biology and Biological Engineering, South China University of Technology, Guangzhou, China; ^2^Department of Thoracic Surgery, Guangdong Provincial People’s Hospital, Guangdong Academy of Medical Sciences, Guangzhou, China

**Keywords:** tumor microenvironment, single-cell RNA sequencing, pan-cancer, SPP1+ tumor-associated macrophage, intercellular crosstalk network, hypoxia

## Abstract

Hypoxia is a characteristic of tumor microenvironment (TME) and is a major contributor to tumor progression. Yet, subtype identification of tumor-associated non-malignant cells at single-cell resolution and how they influence cancer progression under hypoxia TME remain largely unexplored. Here, we used RNA-seq data of 424,194 single cells from 108 patients to identify the subtypes of cancer cells, stromal cells, and immune cells; to evaluate their hypoxia score; and also to uncover potential interaction signals between these cells *in vivo* across six cancer types. We identified SPP1+ tumor-associated macrophage (TAM) subpopulation potentially enhanced epithelial–mesenchymal transition (EMT) by interaction with cancer cells through paracrine pattern. We prioritized SPP1 as a TAM-secreted factor to act on cancer cells and found a significant enhanced migration phenotype and invasion ability in A549 lung cancer cells induced by recombinant protein SPP1. Besides, prognostic analysis indicated that a higher expression of *SPP1* was found to be related to worse clinical outcome in six cancer types. *SPP1* expression was higher in hypoxia-high macrophages based on single-cell data, which was further validated by an *in vitro* experiment that *SPP1* was upregulated in macrophages under hypoxia-cultured compared with normoxic conditions. Additionally, a differential analysis demonstrated that hypoxia potentially influences extracellular matrix remodeling, glycolysis, and interleukin-10 signal activation in various cancer types. Our work illuminates the clearer underlying mechanism in the intricate interaction between different cell subtypes within hypoxia TME and proposes the guidelines for the development of therapeutic targets specifically for patients with high proportion of SPP1+ TAMs in hypoxic lesions.

## Introduction

Tumor is a complex and heterogeneous ecosystem, composed of various cell types and its surrounding tumor microenvironment (TME). Hypoxia is one characteristic of TME, linked to metabolic reprogramming (Xiao et al., 2019) and increased genomic instability (Bhandari et al., 2019), and promotes cancer progression and drug resistance (Hompland et al., 2021). Experimental and clinical studies suggest that T cells, as well as cancer-associated fibroblasts (CAFs) and (TAMs), play significant roles in cancer development and progression under hypoxia TME. For instance, IL1β-IL1R signaling is involved in the stimulatory effects triggered by hypoxia in breast cancer cells, and CAFs promote cancer progression (Lappano et al., 2020). Galectin-3 expressed and secreted from TAMs induced by hypoxia promotes breast tumor growth (Wang et al., 2020). T cell exhaustion, as a common phenomenon in solid tumors, can be mediated by TME. As T cells infiltrated cholesterol-enriched tumor tissues, it would express high levels of immune checkpoints and become exhausted through increasing endoplasmic reticulum stress (Ma et al., 2019). Therefore, explaining the molecular crosstalk between various cells and changes of cellular compositions to environmental pressure is significant for understanding how cancer develops. However, the interaction between tumor cells and other cells has been obtained using cell culture models in most studies (Chen et al., 2019), which reveals the relationship to a certain extent. Tumor and other cells in cell culture settings are not enough to reflect the true conditions of cancer patients’ lesions. Besides, the molecular interaction between tumors and hypoxia TME remains largely unknown.

Recent studies, including The Cancer Genome Atlas (TCGA) project, have achieved molecular subtyping based on various characteristics and identified immune infiltration by using deconvolution on tissue samples in many cancer types (Thorsson et al., 2019). Although these studies have revealed cell proportion in cancer, it remains unresolved how the cells may interact with others to influence cancer development at a very intuitive data level and cannot effectively dissect the heterogeneity of TME. In addition, the gene expression analysis based on bulk cell population averages may be incomplete to reveal the biological properties between cell types in responses to hypoxia stress. The recently developed single-cell transcriptomic technology has great advantages for distinguishing complex cellular compositions and unravelling cell states in tumor tissues (Xiao et al., 2019). Most single-cell studies have focused on distinguishing exhausted CD8+ T cells, TAMs, and CAFs subtypes and also studied the impact of tumor heterogeneity on the effect of drug treatment in a specific cancer type (Kieffer et al., 2020; Lee et al., 2020). However, the heterogeneity and similarity of molecular interaction between distinct cell subtypes across different cancer types and their functional consequences on cancer-promoting effect remain poorly characterized. Moreover, to our knowledge, the direct role of hypoxia on the biological characteristics of each cell subtype as well as on cellular interaction mode between them has not yet been addressed in pan-cancer.

Here, we use single-cell transcriptomic data covering six cancer types and perform a comprehensive analysis to identify the cell subtypes, evaluate their hypoxia score, and to deduce their possible interrelationships in the complex pan-cancer ecosystem landscape. We further identify specific ligand–receptor pairs involved in regulating tumorigenesis and identify specific macrophage subpopulations co-occurring in multiple cancers as key roles linking to poor prognosis and tumor malignancy. Our study illuminates the nature of interactions between cancer cells and the TME and proposes the guidelines for the development of novel therapeutic interventions by targeting hypoxia and cellular crosstalk triggered by hypoxia.

## Materials and Methods

### Data Collection

The single-cell gene expression matrices in the present study were retrieved from the following database: pan-cancer TME blueprint^1^ [including the data of breast cancer (BC), colorectal cancer (CRC), lung cancer (LC), and ovarian cancer (OV; Qian et al., 2020)]; Gene Expression Omnibus [accession numbers: GSE13246, GSE132257, GSE144735 (Lee et al., 2020), and GSE144240 (Ji et al., 2020), including the data of CRC and squamous skin cancer (SCC)]; and Genome Sequence Archive (project number: PRJCA001063) (Peng et al., 2019), including the data of pancreatic ductal cancer (PDAC). Level 3 RNA-seq data and clinical data were downloaded from The Cancer Genome Atlas (TCGA) database^2^. Moreover, the microarray sequencing data of macrophages in hypoxic culture was obtained in GEO database (accession number: GSE4630) (Boström et al., 2006).

### Single-Cell RNA-Seq Data Processing

The raw gene expression matrices were processed using Seurat (v3.2.0) R toolkit. The following quality control steps were applied: (1) genes expressed by <50 cells were not considered and (2) cells that had either fewer than 800 (low-quality cells), over 6,000 expressed genes (possible doublets or multiplets), or over 10% of reads mapping to mitochondrial RNA were filtered out. The sample and remaining cell number in each cancer type is listed in Supplementary Table 1. We obtained the S and G2/M phase score of each cell using the CellCycleScoring function, then normalized the gene expression matrices and regressed out confounding factors such as cell cycle, mitochondrial gene percentage, and total UMI counts using the SCTransform wrapper in Seurat. We constructed principal components (PCs) using highly variable genes generated in the former steps, then selected the first 30 PCs for graph-based clustering with functions FindNeighbors and FindClusters in Seurat. To obtain major cell clusters, the resolution parameter of FindClusters function was set to a small value; to obtain subclusters, we extracted the data of major cell types and reperformed RunPCA, FindNeighbors, and FindClusters. The resolution for each cluster and subcluster analysis is presented in Supplementary Table 2. For visualization of clustering analysis, we performed t-distributed stochastic neighbor embedding (t-SNE) using RunTSNE function in Seurat. As the CRC samples are from different platforms, to increase the accuracy of cell-type designation, we jointly applied a canonical correlation analysis (CCA) before cell-type identification.

We discriminated differentially expressed genes (DEGs) based on Wilcoxon rank-sum test and Model-based Analysis of Single-cell Transcriptomics (MAST) using the Seurat function FindAllMarkers; each cluster was compared to the union of the rest clusters. Genes with a *P*-value < 0.05 were considered as DEGs detected by both Wilcoxon and MAST methods.

### Cell Type and Subtype Annotation

The clusters and subclusters were annotated based on the top-ranking DEGs among the canonical marker genes known from previous studies and literatures. To improve the accuracy of the annotation, we implemented reference-based cell type annotation with SingleR (v1.4.0) and celldex (v1.1.0) R package. Highly expressed markers in each cluster were identified for specific T/NK cells, fibroblasts, myeloid cells, mast cells, endothelial cells, B/plasma cells, and epithelial cells (Supplementary Table 2). To facilitate the identification of numerous cell types, each subcluster was labeled according to the sequence of cells in the cluster tag. Subclusters 0, 1, 2, 3, and 4 of stromal cells were labeled FS1, FS2, FS3, FS4, and FS5 in each cancer type. Subclusters 0, 1, 2, 3, and 4 of cancer cells were labeled CS1, CS2, CS3, CS4, and CS5 in each cancer type. Subclusters 0, 1, 2, 3, and 4 of macrophages/monocytes/dendritic cells were labeled M-S1/Mon-S1/DC-S1, M-S2/Mon-S2/DC-S2, M-S3/Mon-S3/DC-S3, M-S4/Mon-S4/DC-S4, and M-S5/Mon-S5/DC-S5 in each cancer type. Subclusters 0, 1, 2, 3, and 4 of CD8 T cells/CD4 T cells/natural killer cells were labeled CD8-S1/CD4-S1/NK-S1, CD8-S2/CD4-S2/NK-S2, CD8-S3/CD4-S3/NK-S3, CD8-S4/CD4-S4/NK-S4, and CD8-S5/CD4-S5/NK-S5 in each cancer type. Detailed information of cluster including subcluster annotation and cell type markers used in this pipeline are addressed in Supplementary Table 2. In some subclusters, we also found few cells expressing markers from other cell types, which we define as unknown clusters and were removed from further analysis.

### Evaluation of Developmental Trajectory of Myeloid Cells

In order to reveal the cell state transitions, we constructed cell trajectory for monocytes and macrophages using Monocle (v2.18.0) R package (Trapnell et al., 2014). We first excluded dendritic cell clusters from myeloid cells, then substituted Monocle variable genes with the union of DEGs in each subcluster. Dimensional reduction and cell ordering were performed using reduceDimension and orderCells function. The myeloid cells’ cell differentiation trajectory was deduced with the default parameters of Monocle after dimension reduction and cell ordering.

### Definition of Gene Signature Scores Involved in Cell-Specific Function

To make a comparison with the transcriptional signatures of tumor cells, we used the hallmark gene sets from MsigDB^3^ to define cell characteristics by calculating gene set variation analysis (GSVA) score (Hänzelmann et al., 2013). GSVA scores of gene signatures (CAF related, M1/M2 macrophages, pro-inflammatory, anti-inflammatory, etc.) were obtained from previous researches (Azizi et al., 2018; Chen and Song, 2019) to distinguish the features of each cluster in fibroblasts and myeloid cells, respectively. Hypoxia and glycolysis scores were also calculated by GSVA using gene signatures (Wei et al., 2020) across cells and samples in each cancer type. The cytotoxicity and exhaustion activity scores were defined as described in a previous study (Guo et al., 2018). All gene signatures are listed in Supplementary Table 3.

### Gene Signatures of SPP1+ Tumor-Associated Macrophage Cluster

Specific gene signatures of SPP1+ TAM clusters were identified by performing a differential analysis (overlap of Wilcoxon rank-sum test and MAST) between myeloid cell clusters in CRC, LC, and SCC. Differentially expressed genes between clusters (one cluster vs. all other clusters) with an adjusted *P*-value < 0.05 were selected. We used the DEGs in SPP1+ cluster from CRC, LC, and SCC for detecting if these genes were expressed specifically in SPP1+ cluster, and we excluded the genes if it showed an expression level higher than 1 in more than 10% of tumor cells or fibroblasts in CRC, LC, or SCC, respectively. Then, the overlapped genes between three cancer types were defined as SPP1+ TAM signature (Supplementary Table 3). SPP1+ TAM signature score was calculated in bulk RNA-seq data as described above.

### Cell–Cell Interaction Analysis

In order to reveal the molecular mechanism of crosstalk between cells in TME, CellPhoneDB (Efremova et al., 2020) (v2.1.4) was used to calculate ligand–receptor interaction scores in each cell subcluster. This method infers the potential interaction strength between two cell subclusters based on gene expression level and provides the significance through permutation test (1,000 times). To identify biologically relevant interactions, only receptors and ligands expressed in more than a 10% threshold of the cells in the specific cluster were considered for the analysis; log-normalized gene expression matrices were input to CellPhoneDB and ran with the statistical method. We prioritized interactions that were highly enriched between different cell types based on the number of significant pairs, then manually selected biologically relevant pairs by considering the *P*-value (*P*-value < 0.05) and mean expression of the average ligand and receptor level in the present clusters.

### Survival Analysis

The samples were grouped into high and low groups according to the specific gene expression, signature score, or percentage of particular cell types by the median values. Macrophage fractions were estimated by CIBERSORT^4^ with default parameters to eradicate the effects of different cell proportions.

For *SPP1* expression, we performed survival analysis using the top and bottom 50% expression as high and low groups. For *SPP1* expression and TAM proportion, the samples with top and bottom 50% *SPP1* expression and TAM proportion were defined as high and low groups, respectively. The R package “survival” was used to perform the overall survival analysis and produce Kaplan–Meier survival plots. HR and the 95% CI were generated using Cox proportional hazards models.

### Cell Culture, RNA Isolation, and qPCR

The human A549 lung cancer cells were cultured in RPMI-1640 replenished with 10% fetal bovine serum (FBS). All cells were cultured at 37°C in a humidified 5% CO_2_ incubator. The digested cells were counted and inoculated in six-well plates until cell attachment, and then, cells were cultured in a medium added with 100 ng/ml recombinant TNFSF12 (R&D Systems) or 200 ng/ml recombinant SPP1 (R&D Systems), respectively. After 48-h culture, the total RNA was isolated from cells using TRIzol reagent (Magen) according to the manufacturer’s protocol. Reverse-transcribed complementary DNA was synthesized using the Evo-M-MLV RT Kit (AG11705, Accurate Biotechnology). qRT-PCR was performed using the Applied Biosystems QuantStudio 1 Real-Time PCR System (Thermo Fisher) and the PowerUp^TM^ SYBR Green Mix (Thermo Fisher). The fold-change in the expression of target genes was calculated by the 2^–ΔΔ*Ct*^ method. The primer sequence is listed in Supplementary Table 4.

### Wound Healing Assay

We conducted a wound healing assay based on the description of a previous research (Grada et al., 2017). The dissociated cells by trypsin were counted (8 × 10^5^) and inoculated in six-well plates. The cells were cultured until a 90–100% fused cell monolayer formed after 24 h. We then scratched the cells in the fused monolayer with a pipette tip causing an experimental injury and created a linear thin scratch “wound.” Subsequently, cells were cultured in FBS-free medium treated with 100 ng/ml recombinant TNFSF12 (R&D Systems) or 200 ng/ml recombinant SPP1 (R&D Systems), respectively. The wound healing was observed, and images were photographed in 8–15 fields of view that were randomly selected under the MF53-N inverted microscope (MSHOT) in 24 and 48 h. We did three biological repeat experiments. Finally, images of healing were measured and analyzed using ImageJ software (National Institutes of Health).

### Cell Invasion Assay

Matrigel (BD) was diluted by FBS-free 1640 medium and coated on Transwell membrane filter inserts (Corning) to enable analysis of cell invasion. The dissociated cells by trypsin cells were washed by PBS for three times and resuspended by FBS-free 1640 medium. A 200-μl cell suspension with 1 × 10^5^ cells treated with 100 ng/ml recombinant TNFSF12 or 200 ng/ml recombinant SPP1 was inoculated in the upper chamber, respectively, and 700 μl 1640 medium with 10% FBS was added to the lower chamber and cultured at 37°C in a 5% CO_2_ environment. After 24 h, the upper chamber was washed with PBS, and cells were fixed with methanol for 30 min then dyed with 5% crystal violet for 30 min. The images were photographed in five fields of view that were randomly selected under the MF53-N inverted microscope (MSHOT). We did three biological repeat experiments. Finally, images of invasive cells were measured and analyzed using ImageJ software (National Institutes of Health).

### Cell Viability Assays

Logarithmically growing cells were plated into a 96-well plate at a density of 1 × 10^3^ cells/well and 100 ng/ml recombinant TNFSF12 (R&D Systems) or 200 ng/ml recombinant SPP1 (R&D Systems) was added after 12 h and then incubated for 0, 24, 48, 72, 96, and 120 h. Recombinant protein was added to cultured media once, and the media were not changed for 24, 48, 72, 96, and 120 h. Before proliferation ability was detected, 10 μl of Cell Counting Kit-8 (CCK8) solution (GlpBio) was added to the cultures. After incubation for 1 h in a humidified incubator containing 5% CO_2_ at 37°C, absorbance was detected at 450 nm.

### Hypoxia Treatment of THP-1-Derived Macrophages

Human monocyte cell THP-1 were cultured in RPMI-1640 replenished with 10% FBS, and 100 U/ml penicillin and 100 mg/ml streptomycin were added. All cells were cultured at 37°C in a humidified 5% CO_2_ incubator. For cell differentiation, THP-1 monocytes were seeded at 8 × 10^5^ cells/well in six-well plates and directly differentiated into macrophages by 24-h incubation with 100 ng/ml phorbol 12-myristate 13-acetate (PMA, Sigma), followed by a 24-h rest period in complete RPMI-1640 medium without PMA. At the end of 48 h, THP-1 macrophages were used as M0 macrophages. The total RNA was immediately isolated from cells using TRIzol reagent (Magen) according to the manufacturer’s protocol.

THP-1-derived M0 macrophages were cultured in a six-well plate and incubated at 37°C under normoxia (21% O_2_ and 5% CO_2_) or hypoxia (1% O_2_, 5% CO_2_, and balanced N_2_) in a hypoxic environment chamber (Maworde), respectively, for 24 h. The total RNA was immediately isolated from cells using TRIzol reagent (Magen) according to the manufacturer’s protocol.

### Statistical Analysis

All statistical analyses and graphical representation of data were performed in the R environment (version 4.0.3) or using GraphPad Prism software (version 7.0). The correlation analysis including gene expression, gene signature score, and cell proportion between the two groups used in this study was based on Spearman correlation. For the cell subtype abundance correlation matrix, we defined the number ratio of cell subtype to the belonging major cell type as the relative abundance of each cell subtype, then computed the Spearman correlation coefficient between the relative abundance of each cell subtype in six cancer types.

For the difference analysis between groups, we used Wilcoxon rank-sum test throughout the analysis on single-cell and bulk RNA-seq data. For the differential gene analysis between hypoxia-high and -low groups in TCGA, we used edgeR (Robinson et al., 2010) to get DEGs and used Metascape (Zhou et al., 2019) for gene enrichment analysis. For the cell experiment, the unpaired two-tailed *t*-test was used to compare the difference between experimental groups and control groups.

## Results

### Global Cellular Landscape of Six Cancer Types Revealed by scRNA-Seq Analysis

**FIGURE 1:**
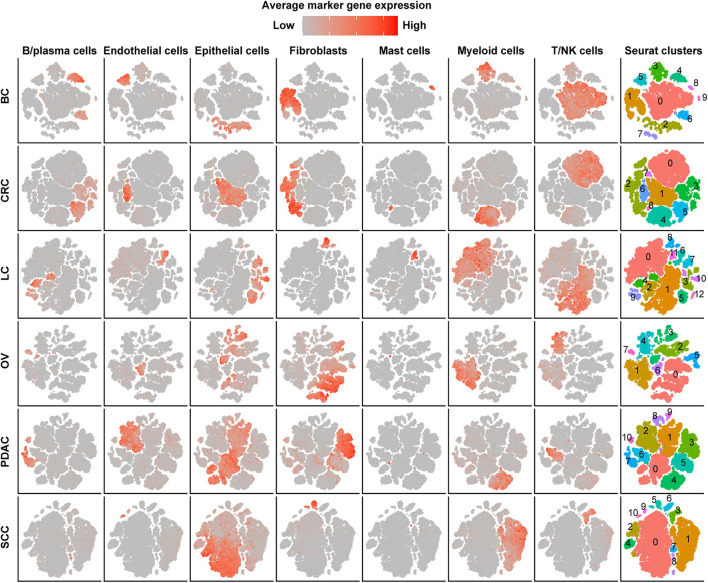
Cell-type identification in pan-cancer. t-SNE map of single cells from cancer tissues to visualize cell-type clusters based on the expression of known marker genes [T/NK cells (*CD2*, *CD3D*, *CD3E*, *CD3G*, *FGFBP2*, *XCL1*, *FCGR3A*, *KLRD1*, and *KLRF1*), fibroblasts (*FAP*, *PDPN*, *COL1A2*, *DCN*, *COL3A1*, *COL6A1*, and *LUM*), myeloid cells (*CD14*, *CD16*, and *CD68*), mast cells (*CMA1*, *MS4A2*, *TPSAB1*, *TPSB2*, and *CPA3*), endothelial cells (*PECAM1*, *VWF*, *ENG*, *PLVAP*, and *SELE*), B/plasma cells (*SLAMF7*, *CD79A*, *BLNK*, *FCRL5*, and *CD79A*), and epithelial cells (*EPCAM*, *KRT19*, *KRT7*, *KRT18*, *KRT1*, *DMKN*, and *KRT6C*)].

After strict quality control (QC) and filtration, we collected 25,318, 15,347, 6,019, 14,991, and 21,447 single cells originating from normal tissues; 57,486, 32,509, 17,732, 40,940, and 25,772 tumor-derived cells in CRC, LC, OV, PDAC, and SCC, respectively; and 24,160 tumor-derived cells in BC. We divided all cells for each cancer type into 6–10 major clusters and identified epithelial cells, stromal cells (fibroblasts, pericytes, and endothelial cells), and immune cells (T/NK cells, B/plasma cells, myeloid, and mast cells) as the major cell types (Figure 1). We observed that the cell proportion of each cell type was different among cancer types. T/NK cells were only 4% in SCC, 7% in PDAC, and 10% in OV, while they were 32% in CRC, 42% in LC, and 45% in BC (Supplementary Figure 1A and Supplementary Table 1). Besides, the proportion of cells in each patient also varied, indicating intertumoral heterogeneity (Supplementary Figure 1B).

### Hypoxia Score of Cell Subtypes in Stromal Cells, Myeloid Cells, and T Cells

**FIGURE 2:**
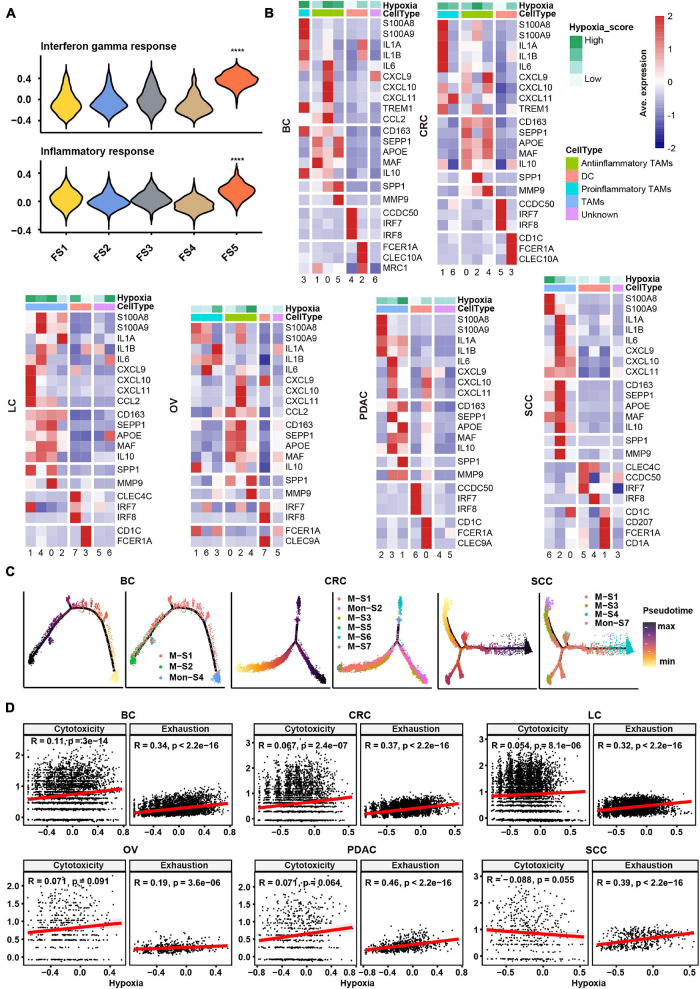
Subtype classification and characteristics of non-malignant cells across cancer types. **(A)** Hallmark gene set scores for interferon gamma response (top) and inflammatory response (bottom), computed for all fibroblast clusters in BC. FS1, FS2, FS3, FS4, and FS5 are subclusters 0, 1, 2, 3, and 4 in stromal cells, see *Materials and Methods* and Supplementary Figure 2A. ****p ≤ 0.0001, two-sided Wilcoxon rank sum test. **(B)** Heatmap of *Z*-score-normalized log_2_ (count+ 1) expression of canonical marker genes for myeloid cells. The color of the square on the top map indicates the average hypoxia score for each myeloid cell cluster (low to high, light green to green). **(C)** The branched trajectory of myeloid cell state transition in cancer (BC), colorectal cancer (CRC), and squamous skin cancer (SCC) inferred by Monocle 2. Each dot corresponds to one single cell, colored according to its cluster label. Subclusters 0, 1, 2, 3, and 4 of macrophages/monocytes were labeled M-S1/Mon-S1, M-S2/Mon-S2, M-S3/Mon-S3, M-S4/Mon-S4, and M-S5/Mon-S5 in each cancer type, see *Materials and Methods*. **(D)** Correlation scatter plot between gene set variation analysis (GSVA) scores of hypoxia, cytotoxicity, or exhaustion.

In order to evaluate the hypoxia level of these major cell types in the TME, we performed a subcluster analysis on cells from cancer tissues and calculated hypoxia score in each subtype across cancer types. Subclustering of stromal cells mainly revealed three broad classes: pericytes, myofibroblasts, and fibroblasts (Supplementary Figure 2A). As there were few fibroblasts (710 cells) in SCC, we failed to subcluster stromal cells in this cancer, and the remaining studies of stromal cells focused on the other five cancer types. We named fibroblast clusters in order in the form of labels (e.g., FS1 and FS2 are clusters 0 and 1, respectively). According to the markers from previous studies (Costa et al., 2018; Kieffer et al., 2020) and significant up-regulated genes in each cluster, we then termed fibroblasts into collagen-related CAFs, chemokine-related CAFs, and interleukin (IL) signal-related CAFs in specific cancer types. IL signal-related CAFs [FS5 (cluster 4) in BC] up-expressed inflammatory signatures such as interferon response and inflammatory response in BC (Figure 2A). Collagen-related CAFs [FS1 (cluster 0) in six cancer types] exhibited the highest extracellular matrix (ECM) remodeling score (Supplementary Figure 2B). However, there was no consistent trend among CAFs in hypoxia scores across cancer types.

Myeloid cells were investigated in two aspects, including identification of subtypes and evaluation inflammatory features. Using conventional marker genes, we identified dendritic cells, monocytes, and macrophages and found the common subsets across all cancer types (Supplementary Figure 2C). We distinguished the pro-inflammatory and anti-inflammatory monocyte/macrophages according to the markers/gene sets referenced in previous studies (Azizi et al., 2018; Figure 2B and Supplementary Figure 3A). However, some specific TAMs had a mixed phenotype, expressing both pro-inflammatory and anti-inflammatory signatures as well as M1 and M2 gene signatures (Figure 2B and Supplementary Figures 2C, 3A), consistent with previous studies (Lee et al., 2020). The noteworthy phenomenon was that *SPP1* was expressed higher in one subtype, such as M-S1 (cluster 0) in BC; M-S3 (cluster 2) in CRC; M-S1 (cluster 0) and M-S2 (cluster 1) in LC; M-S1 (cluster 0) and M-S5 (cluster 4) in OV; M-S2 (cluster 1) in PDAC; and M-S3 (cluster 2) in SCC, which were universal across six cancer types (Figure 2B and Supplementary Table 2). We named these subtypes as SPP1+ TAMs and found matrix metallopeptidase 9 (*MMP9*), associated with ECM remodeling, was also highly expressed in SPP1+ TAMs (Figure 2B). It was worth noting that the hypoxia score was higher in SPP1+ TAMs compared with other subtypes (Figure 2B). Given the above characteristics of SPP1+ TAMs, they might play a central role in tumor progress under the influence of hypoxia TME.

As TAMs can be either tissue-resident or monocyte-derived (Yona et al., 2013), a cell trajectory analysis was employed to explore the lineage trajectories of the macrophage and monocyte populations (Figure 2C and Supplementary Figure 3B). The Monocle trajectory analysis suggested that some TAM clusters could be monocyte derived, such as M-S1 and M-S2 derived from Mon-S4 in BC and M-S1, M-S3, and M-S4 derived from Mon-S7 in SCC, while others appeared to be tissue-resident macrophages in origin, such as M-S1 in CRC (Figure 2C). Taken together, our findings illustrate that TAMs may undergo different transcriptional reprogramming like the pro- and anti-inflammatory differentiation axis and also suggest a more complex phenotype of TAMs in the TME across different cancer types.

Subclustering of T/NK cells led to the identification of CD4+ T cells, CD8+ T cells, and NK cells (Supplementary Figure 3C). We intended to identify the exhaustion status of CD8+ T cells from the gene expression of key inhibitory receptors (*PDCD1*, *TIGIT*, *HAVCR2*, *LAG3*, and *CTLA4*) (Supplementary Figure 3C). However, cells expressing exhaustion genes also highly express cytotoxicity markers (*GZMB* and *IFNG*) in CD8+ T cells, which further confirmed that one specific subcluster highly exhibited both cytotoxicity score and exhaustion score (Supplementary Figure 3D). As shown in Supplementary Figure 3D, CD8-S3 (cluster 4) in CRC expressed both higher cytotoxicity and exhaustion scores compared with other clusters. This observation appeared to an activation-dependent exhaustion expression program similar to the previous scRNA-seq study (Guo et al., 2018). Unexpectedly, the exhausted CD4+ T cells [CD4-S5 (cluster 4) in BC; CD4-S9 (cluster 8) in CRC; CD4-S6 (cluster 5) in LC; CD4-S4 (cluster 3) in PDAC; and CD4-S4 (cluster 3) in SCC] were distinguished across five cancer types. As a previous study (Scharping et al., 2021) showed that T cell exhaustion was driven under hypoxic environment, it was suggested that there may be an association between hypoxia and exhaustion. We then preformed a correlation analysis and found that hypoxia score was highly correlated with exhaustion score but not cytotoxicity score in T cells across six cancer types (Figure 2D).

### Transcriptional Heterogeneity of Malignant Cells and the Association With Hypoxia

**FIGURE 3:**
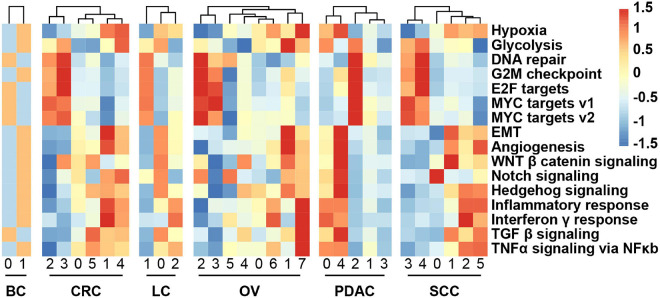
Heatmap showing different hallmark gene sets enriched in the cancer cell subclusters by GSVA, colored by *Z*-score-transformed mean GSVA scores.

We obtained the malignant epithelial cell subclusters and DEGs of each subcluster. We next explored how expression states varied among different cancer cells within the same cancer type, and the GSVA reflecting the activity of cancer-related hallmark pathways was applied. GSVA distribution of some subclusters revealed a significant enrichment of genes related to epithelial–mesenchymal transition (EMT) and angiogenesis, while some subclusters were highly enriched in cell cycle-related hallmarks: E2F/MYC targets and G2M checkpoint, indicating intratumoral heterogeneity (Figure 3). Notably, it was observed that subclusters with high hypoxia was the same as the subclusters with EMT program.

### Crosstalk Between Stromal and Myeloid Cells and Cancer Cells

To decipher the molecular associations underlying cell–cell interactions, we constructed a cellular communication network between different cell subtypes using potential ligand–receptor (L–R) pair interactions (Supplementary Figure 4A and Supplementary Table 5). Importantly, the numbers of interaction between cancer cells and myeloid cells were predicted to be the most universal within the cellular network across six cancer types (Supplementary Figure 4A). Besides, we next analyzed whether there was any correlation between the respective proportions between these subclusters across patients and found some co-occurring cell subclusters (Supplementary Figure 4B and Supplementary Table 6), such as proportions between some fibroblast and cancer cell subclusters that were correlated in BC and OV.

**FIGURE 4:**
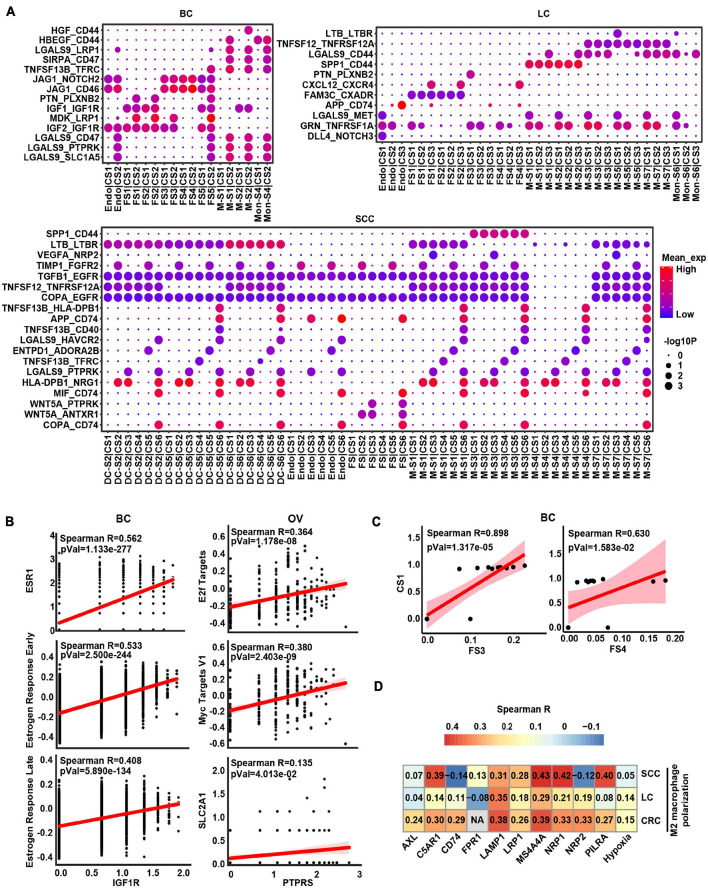
The intercellular interactions between non-malignant cells and cancer cells. **(A)** Significant ligand–receptor genes accounting for specific intercellular interactions in BC, LC, and SCC. *P*-values are indicated by circle size. The means of the average expression level of interacting molecule (ligand or receptor genes) 1 in cluster 1 and interacting molecule 2 in cluster 2 are indicated by color. Subclusters 0, 1, 2, 3, and 4 of stroma cells were labeled FS1, FS2, FS3, FS4, and FS5 in each cancer type. Subclusters 0, 1, 2, 3, and 4 of cancer cells were labeled CS1, CS2, CS3, CS4, and CS5 in each cancer type. Subclusters 0, 1, 2, 3, and 4 of macrophages/monocytes/dendritic cells were labeled M-S1/Mon-S1/DC-S1, M-S2/Mon-S2/DC-S2, M-S3/Mon-S3/DC-S3, M-S4/Mon-S4/DC-S4, and M-S5/Mon-S5/DC-S5 in each cancer type. Endo is endothelial cells. **(B)** Correlation scatter plot between main receptors expressed on cancer cells and specific pathways as well as genes in BC and OV. **(C)** Correlation between proportional changes in specific stromal cell cluster and cancer cell cluster in BC. **(D)** Heatmap depicts the correlations between M2 macrophage polarization and hypoxia as well as main receptors expressed on macrophage.

Given that crosstalk between cancer cells and myeloid cells as well as stromal cells was predicted to be universal, we focused an analysis on interactions between these cell types and interrogated how they influenced each other in a particular way to promote cancer progression (Figure 4A and Supplementary Figure 5). In BC and OV, the stromal cells were the widespread cell types interacting with cancer cells (Figure 4A and Supplementary Figure 5). Insulin-like growth factor 1 (*IGF1*) may be secreted by stromal cells to regulate cancer cell growth through binding their receptors on the cancer cell. Insulin-like growth factor 1 receptor (*IGF1R*) gene expressed by cancer cells was highly associated with estrogen response signatures in BC, which may demonstrate that the binding effect of IGF1 on IGF1R as well as activating estrogen signaling enhanced cancer growth (Figure 4B). Protein tyrosine phosphatase receptor type S (*PTPRS*) was highly expressed in OV cancer cells and correlated with MYC/E2F targets, indicating that pleiotrophin (*PTN*) was secreted by fibroblasts binding to its receptor to promote the cancer cell growth (Figure 4B). Correspondingly, the expression of *IGF1R* and *PTPRS* was positively correlated with estrogen response-related gene *ESR1* and MYC target gene *SLC2A1* in BC and OV, respectively, (Figure 4B). Furthermore, the proportion of cancer cell subcluster [CS1 (cluster 0)] and stroma cell subcluster (FS3 and FS4) also displayed positive correlations in BC (Figure 4C). These results showed that fibroblasts potentially promoted tumor cell proliferation by expressing and secreting different growth factors.

Obviously, some L–R pairs between myeloid cell and cancer cell interaction were universal. For example, *TNFSF12-TNFRSF12A* and *SPP1-CD44* were shown in LC, CRC, and SCC (Figure 4A and Supplementary Figure 5), indicating that myeloid cells might express and secrete TNFSF12 and SPP1, signaling to their receptors TNFRSF12A and CD44 on cancer cells, respectively. Conversely, we also predicted the interaction between ligand on cancer cells and receptor on myeloid cells. The result showed TAMs would receive activated signals from cancer cells through *GAS6-AXL*, *RPS19-C5AR1*, *FAM3C-LAMP1*, *CD47-SIRPA*, and *VEGFA-NRP1/NRP2* L–R pairs in TME (Supplementary Table 5). Besides, these receptors of TAMs were correlated with M2 macrophage polarization (Figure 4D), suggesting cancer cells could possibly serve as the potential source of the ligand for activation of M2-like TAMs in TME. It is worth noting that hypoxia score also had a positive correlation with M2 macrophage polarization, which could speculate that hypoxia is a potential factor affecting cell communication (Figure 4D). Overall, these results indicated that tumor cells and macrophages formed a positive feedback interaction *via* ligand–receptor signaling in the TME.

### Tumor-Associated Macrophages Potentially Promote Epithelial–Mesenchymal Transition of Cancer Cells

**FIGURE 5:**
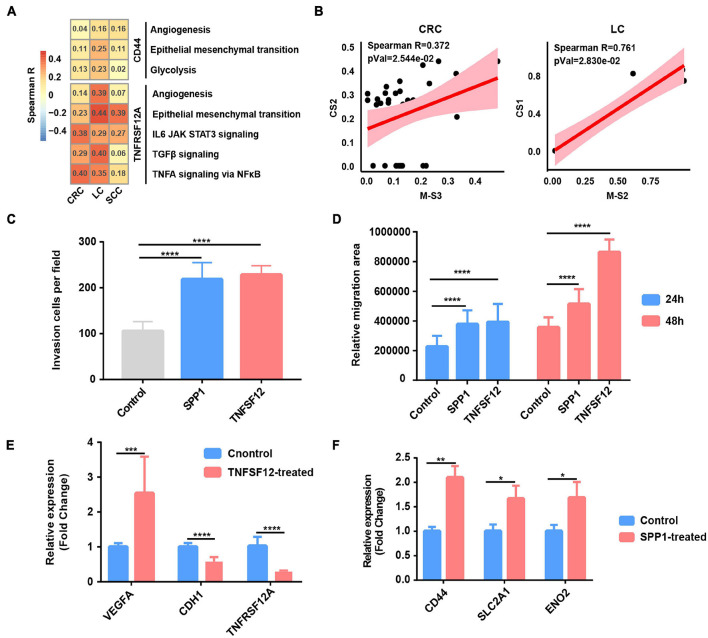
Tumor-associated macrophages (TAMs) promote glycolysis, invasion, and migration phenotype of cancer cells. **(A)** Heatmap depicts the correlation between *CD44* or *TNFRSF12A* expression and hallmark signatures scores in cancer cells. **(B)** The correlation between proportional changes in SPP1+ TAM cluster (M-S3 and M-S2 in CRC and LC, respectively) and epithelial–mesenchymal transition (EMT)-related cancer cells (CS2 and CS1 in CRC and LC, respectively). **(C)** Box plot shows relative invasion cells per field of A549 cells treated by recombinant protein SPP1 and TNFSF12 for 24 and 48 h. ****p ≤ 0.0001, two-sided unpaired *t*-test. **(D)** Box plot shows relative migration area of A549 cells treated by recombinant protein SPP1 and TNFSF12 for 24 and 48 h. ****p ≤ 0.0001, two-sided unpaired *t*-test. **(E)** Relative mRNA expression of specific genes (*VEGFA*, *CDH1*, and *TNFRSF12A*) in A549 lung cancer cells exposed to TNFSF12 for 48 h. **p ≤ 0.01, two-sided unpaired *t*-test. **(F)** Relative mRNA expression of specific genes (*CD44*, *SLC2A1*, and *ENO2*) in A549 lung cancer cells exposed to SPP1 for 48 h. *p ≤ 0.05; **p ≤ 0.01, two-sided unpaired *t*-test.

In order to study the specific effect of macrophages on tumor cells through the common L–R pairs (*TNFSF12–TNFRSF12A* and *SPP1–CD44*), we further calculated the correlation between the *TNFRSF12A* or *CD44* expression and hallmark signature scores in cancer cells. The results revealed that angiogenesis, glycolysis, and EMT were the biological process most correlated with *CD44* expression, while the TNFα signaling *via* NFκB, angiogenesis, IL6_JAK_STAT3 pathway, and EMT were correlated with *TNFRSF12A* in cancer cells across CRC, LC, and SCC (Figure 5A and Supplementary Table 7). As *SPP1* was mainly expressed in macrophage (Supplementary Figure 6A), we further detected that the relative abundances of SPP1+ TAMs [M-S2 (cluster 1) in LC and M-S3 (cluster 2) in CRC] and cancer cell subclusters with high EMT [CS1 (cluster 0) in LC and CS2 (cluster 1) in CRC] were correlated together in LC and CRC, which further strengthens the function of SPP1+ TAMs in promoting EMT (Supplementary Table 2 and Figure 5B).

To evaluate the functional significance of the above key L–R interactions in lung cancer, A549 lung cancer cells were exposed to human recombinant protein SPP1 or TNFSF12 for 24 and 48 h, respectively. Compared with the control, cells exposed to recombinant protein TNFSF12 exhibited significantly enhanced migration and invasion ability but a slightly reduced proliferation of lung cancer cells (Figures 5C–E and Supplementary Figures 6B–D). In line with the phenotypic changes, TNFSF12 treatment led to a decreased expression of the epithelial marker E-cadherin (*CDH1*) (Figure 5E). It is known that VEGF is an NFκB-inducible protein and is one of the most potent angiogenic factors crucial for tumor metastasis (Leung et al., 1989), and qRT-PCR analysis showed that *VEGFA* expression was remarkably upregulated in lung cancer cells with recombinant protein TNFSF12 treatment at 48 h (Figure 5E). However, *TNFRSF12A* expression was reduced after TNFSF12 treatment, which needs a further extensive study to investigate its molecular mechanism. We observed an enhancement of cell migration and invasive behavior in lung cancer cells induced after SPP1 treatment (Figures 5C,D and Supplementary Figures 6B,C). By performing qRT-PCR, we observed that cancer cells exposed to SPP1 exhibited a significantly increased gene expression in EMT-related molecule *CD44* and glycolytic genes including *SLC2A1* and *ENO2* at 48 h (Figure 5F).

To further verify our single-cell analysis and *in vitro* experiment results, we then extended our analysis to TCGA LUAD database and found that *TNFSF12* expression was positively correlated with EMT score, but negatively correlated with proliferation score (Supplementary Figure 6E). There was a strong correlation between *SPP1* expression and glycolysis, EMT score, and related genes (Supplementary Figure 6E), which further reinforced that SPP1+ TAM-derived SPP1 might participate in facilitating glycolysis and EMT in lung cancer cells. Taken together, our results of the paracrine interactions analysis and *in vitro* experiment highlight the cancer-promoting role of SPP1 and TNFSF12 signaling.

### SPP1 Is Upregulated in Hypoxia Tumor Microenvironment and Associated With Poor Prognosis

**FIGURE 6:**
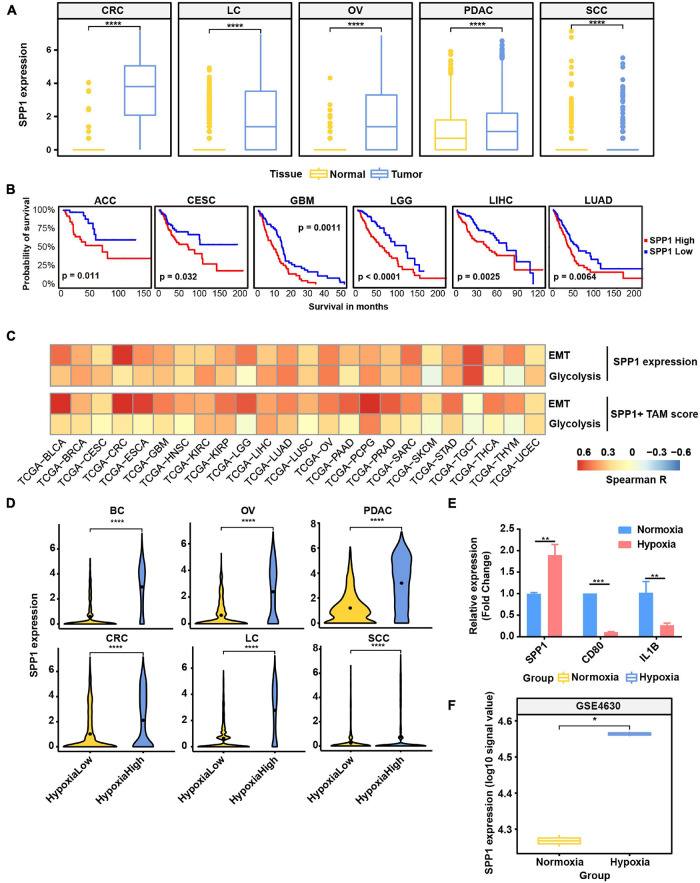
*SPP1* is related to poor prognosis and upregulated in hypoxia tumor microenvironment (TME) to promote malignant phenotype of cancer. **(A)** The expression of *SPP1* in macrophage from tumor and normal samples. ****p ≤ 0.0001, two-sided Wilcoxon rank sum test. **(B)** The Kaplan–Meier overall survival curves of The Cancer Genome Atlas (TCGA) patients grouped by gene expression of *SPP1*. **(C)** The correlation between *SPP1* expression and SPP1+ TAM signature score with glycolysis score and EMT score in TCGA cancer samples. **(D)** Violin plot shows the *SPP1* expression in hypoxia-high and -low macrophages in six cancer types. **(E)** Relative mRNA expression of *SPP1* and M1 marker genes in THP-1-derived macrophage exposed to hypoxia (1% O_2_) for 24 h. **(F)** The expression of *SPP1* in macrophage exposed to hypoxia and normoxia from GSE4630 data.

As SPP1+ TAMs were revealed to harbor higher hypoxia score (Figure 2B) and co-occur with EMT cancer cells (Figure 5B), we focused on exploring the functions of SPP1 and SPP1+ TAMs. *SPP1* was upregulated in macrophage derived from tumor samples compared with that from normal tissues (Figure 6A); we reasoned that *SPP1* was a specific TME-induced expression program in TAMs. These findings were further confirmed by TCGA cancer samples, which showed that compared with adjacent normal tissues, a much higher expression of *SPP1* in tumor tissues was observed in corresponding cancer types (Supplementary Figure 7A). Meanwhile, PDAC and LC were found to harbor a higher proportion (>50%) of SPP1+ TAMs (Supplementary Figure 7B). Using clinical data collected from the TCGA project, we confirmed that patients with a higher level of *SPP1* gene expression showed worse prognosis in six cancer types, including lung cancer studied in this study (Figure 6B), and a higher proportion of SPP1+ TAMs was also associated with a worse clinical outcome (Supplementary Figure 7C), suggesting the clinical impact of *SPP1* and SPP1+ TAMs in cancer.

As showed above, *SPP1* and *MMP9* were co-expressed in SPP1+ TAMs (Figure 2B); we reasoned that SPP1+ TAMs might participate in ECM remodeling. Using the ECM remodeling signatures, we assessed the functional phenotypes of each macrophage subtypes across different cancer types. As expected, the SPP1+ TAMs showed preferential ECM remodeling (Supplementary Figure 7D), while other TAMs exhibited lower performance. Due to the role of ECM remodeling in cancer glycolysis, angiogenesis, and metastasis, we investigated the association between SPP1+ TAM signature, SPP1 expression with glycolysis, and EMT program, respectively, and found that there was a positive correlation between them in multiple cancer types (Figure 6C). These results may underscore the potential cancer-promoting role of SPP1+ TAMs in complex TME.

As showed in Figure 6D and Supplementary Figure 7E, *SPP1* expression was higher in hypoxia-high macrophages, and the hypoxia score was higher in SPP1+ TAMs. Consistent with the results in single-cell data, the expression of *SPP1* was higher in hypoxia-high samples than that in low ones (Supplementary Figure 8). To further verify whether *SPP1* expression is directly regulated by hypoxic stress, we performed cell culture experiment and confirmed that *SPP1* expression was significantly upregulated in THP-1-derived macrophages exposed to hypoxic (1% O_2_) than to normoxic (21% O_2_) conditions for 24 h (Supplementary Figures 7F,G and Figure 6E). We observed a higher *SPP1* expression in human mononuclear cell-derived macrophages exposed to hypoxic (1% O_2_) than to normoxic conditions for 24 h, which was further confirmed by an independent GEO dataset (Boström et al., 2006; Figure 6F). Thus, we reasoned that *SPP1* was upregulated, and SPP1+ TAMs were expanded in hypoxia TME, interacting with cancer cells to promote malignant biological characteristics and thus bring poor survival of patients.

### Hypoxia Potentially Affecting the Biological Characteristics and Functions of Different Tumor-Infiltrating Cell Types

**FIGURE 7:**
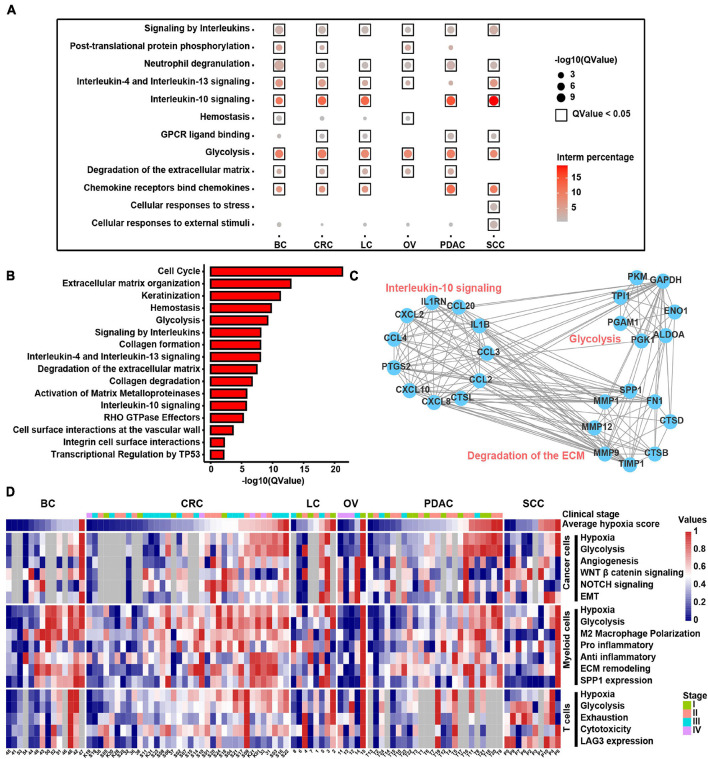
Molecular characteristics of different cell types across samples under the hypoxia TME. **(A)** Enriched Reactome gene sets of upregulated genes in hypoxia-high myeloid cells across six cancer types. **(B)** Enriched Reactome gene sets of upregulated genes in hypoxia-high samples at least in 13 TCGA cancer types. **(C)** The protein–protein interactions among differentially expressed genes (DEGs) upregulated in hypoxia-high myeloid cells. **(D)** Clustered heatmap of 18 features across pan-cancer samples. Samples are arranged from low hypoxia score to high hypoxia score with the color blue to red, respectively. For tumors, the stage is indicated by color. Gray rectangles highlight that there were less than 50 cells in this sample. The values were normalized from 0 to 1 by Minmax.

To discover the hypoxia effect on gene expression spectrum in different cell types, we compared the gene expression of the hypoxia-high and hypoxia-low cells by DEG analysis (1.5-fold difference, adj. *p* < 0.05) coupled with Reactome term enrichment analysis (adj. *p* < 0.01) of DEGs across T cells, fibroblasts, myeloid cells, and cancer cells. As the DEGs were fewer in T cells and fibroblasts, and we mainly focused on myeloid and cancer cells (Supplementary Table 8). Among the DEGs in myeloid cells, *SPP1* and *TIMP1* were the most significantly upregulated genes in hypoxia-high cells across cancers (Supplementary Figure 9A). As shown in Figure 7A, signaling by interleukins including IL4, IL13, and IL10 signals were enriched in hypoxia-high myeloid cells, indicating that immunosuppressive cytokines were activated in hypoxia TME. Besides, degradation of ECM and glycolysis were active in hypoxia-high myeloid cells across six cancer types. However, except glycolysis, the enriched pathways in hypoxia-high cancer cells varied among cancer types, suggesting a tissue-specific response to hypoxia (Supplementary Figure 9B). Moreover, we included 25 TCGA cancer types and performed DEG analysis. From the DEGs, there were 489 genes upregulated in hypoxia-high tumors versus low tumors in more than 13 cancer types (Supplementary Table 7). We found that IL signaling and ECM degradation, as well as glycolysis, were significantly enriched in hypoxia-high tumors (Figure 7B). Biological processes of matrix proteoglycan, like collagen formation, collagen degradation, and integrin cell surface interactions, were also identified in TCGA cancers. Moreover, we found that the DEGs, upregulated in hypoxia myeloid cells across six cancer types, interacted with each other frequently in protein–protein interaction networks (Figure 7C). Taken together, these results suggest that cross-talk among these molecules up-expressing under hypoxia TME, may play critical roles in the development and progression of different cancer types.

In addition, we returned the key hypoxia-related molecular characteristics of above results back to individual samples to further inspect their relationship and distribution and found that most of the characteristics in the individual were consistent with the overall distribution across six cancer types (Figure 7D). For example, the extensive association between hypoxia and glycolysis in different types of cells was observed at the individual level. *SPP1* expression in myeloid cells along with EMT and glycolysis program in cancer cells was higher in hypoxia-high samples. Thus, this analysis provides a theoretical basis for studying the intratumoral heterogeneity and intertumoral consistency within multiple cell types and also provides the clinical guidance value to single patients. In general, the above results show that hypoxia is disclosed to be the major factor to influence the intercellular crosstalk and shows different contributions to each cell types, participating in SPP1+ TAM expansion, ECM remodeling, and interleukin-10 signal activation to accelerate cancer EMT, glycolysis, and angiogenesis.

## Discussion

In the present study, we identified multiple subclusters among different cell types that shape the heterogeneous TME and share consistency across different cancer types. We illustrated the cellular communication landscape between malignant and non-malignant cells and highlighted the reciprocal relationship between them. We distinguished that SPP1+ TAMs, expanded under hypoxia TME, might promote the EMT and glycolysis program of cancer cells and might be related to worse survival in multiple cancer types. This study depicts the comprehensive cellular interaction map of BC, CRC, OV, LC, PDAC, and SCC and provides a framework for future discoveries of molecular and cellular therapeutic targets to block the interactions between cancer cells and TME to inhibit cancer development more thoroughly and effectively.

Sufficient cells in this study enable us to distinguish different macrophage clusters and highlight the SPP1+ TAM subtype, which is activated under the hypoxia TME, and higher *SPP1* expression was linked to poor prognosis in multiple types. Studies have previously shown that stromal SPP1 promotes cancer cell survival and enhances invasion behavior in glioma (Lu et al., 2012), prostate cancer (Pang et al., 2019), and melanoma (Kale et al., 2015), suggesting a direct effect of SPP1 on tumor cells. Besides, a recent study based on single-cell analysis showed that SPP1+ TAMs were associated with tumor angiogenesis in various cancer types (Cheng et al., 2021). Our current study shows that EMT is the biological process in cancer cells most associated with SPP1+ TAMs as revealed by single-cell analysis, and the potential effect of SPP1+ TAMs on cancer cells is further confirmed by TCGA bulk data. The results indicate that SPP1+ TAMs may interact with cancer cells in a paracrine pattern through expressing and secreting SPP1 then binding to cell-surface receptor CD44, consistent with a previous work, showing that CD44 is the receptor of SPP1 to regulate cancer metastasis (Wai and Kuo, 2004). One study indicates that SPP1 activates JNK signaling through a CD44v6-dependent pathway to promote clonogenicity of colorectal cancer cells, and the CD44v6 antibody is able to potently block the activation of JNK induced by SPP1 (Rao et al., 2013). Besides, another study on human optic nerve head astrocytes shows that a CD44-blocking antibody led to a significant increase of metabolic activity caused by SPP1 signaling (Neumann et al., 2014). Our results, combined with previous studies, suggest that the SPP1–CD44 interaction is important for cancer progression. Thus, the identification of SPP1 as an abundant TAM-secreted factor in cancer, coupled with the pro-tumor impact of SPP1, suggests that inhibiting SPP1 at transcriptional or protein level, blocking the interaction between SPP1+ TAMs and cancer cells through targeting SPP1 and CD44, may be an effective clinical strategy for tumor growth and metastasis inhibition. For example, small interfering RNA against SPP1 by intratumoral injection significantly suppressed breast tumor growth and angiogenesis in a mouse model (Cho et al., 2015). Blocking antibodies to SPP1 and its specific receptors CD44 showed an inhibitory role in cancer cell migration. Researchers showed that the blocking antibody targeting CD44 on stromal cells reduced the SPP1-induced breast cancer metastasis (Mi et al., 2011). SPP1-R3 aptamer was used to inactivate SPP1 and disturb surface binding of SPP1 to its cell surface CD44 receptor and mediators of ECM degradation, MMP-2, in human breast cancer cells (Mi et al., 2009). However, although there are a considerable number of therapeutic approaches by targeting SPP1 based on preclinical studies, only a few number of findings translate into clinical practice, and SPP1 inhibitors or combination drug therapy should still be further investigated from bench to clinic (Wei et al., 2017). Thus, future research is needed to elucidate the roles of SPP1 and explore the underlying molecular mechanism of SPP1 in cancer progression.

As hypoxia is one of the key environmental stresses in tumor tissues, resulting in aggressive cancer phenotypes (Haider et al., 2016), we go a step further by analyzing the association between hypoxia and cell characteristics. Although the association between hypoxia and TAMs has been studied by various researches (Henze and Mazzone, 2016), to our knowledge, this is the first study to discover a strong association between *SPP1* expression as well as SPP1+ TAM abundance and hypoxia. In exploring the link between SPP1 and hypoxia, we observed that *SPP1* gene expression was higher in hypoxia samples both in single-cell and tissue samples (Figure 6D; Supplementary Figure 8). *SPP1* expression is also upregulated under hypoxia conditions in cell culture system (Figures 6E,F), which indicated that *SPP1* expression was directly regulated by hypoxia. Disordered glycolysis, as an oncogenic event, is also higher in hypoxia cancer cells, which is consistent with the findings of a single-cell research where glycolysis and hypoxia signature were highly correlated in melanoma and HNSCC cancer cells (Xiao et al., 2019). Conceivably, the cancer EMT and glycolysis program promoted by SPP1+ TAMs may also be accelerated by hypoxia TME, as there is a strong correlation between the abundance of SPP1+ TAMs and EMT, glycolysis, and hypoxia. As reported by a previous work (Colegio et al., 2014) that M2 macrophage polarization is associated with the hypoxia TME and thus promotes tumor growth, we further uncover the specific molecules involved in these processes, including NRP1/NRP2 and LAMP1 expressed on TAMs. Moreover, hypoxia, in the current study, is disclosed to be a factor to influence the intercellular crosstalk, metabolic reprogramming, tumor heterogeneity, SPP1+ TAM expansion, and T cell exhaustion, thus promoting cancer development.

## Conclusion

In summary, our work identifies the significant cell subpopulations and the interactions between them, which may provide a theoretical framework for understanding that tumor heterogeneity and diversity are driven not only by genetic and epigenetic factors but also by a combination of factors, including TME stress and other cell types surrounding tumors. The intercellular interactions suggest a tight molecular relationship between different cell types that may determine the progression and the prognosis in cancer and also encourage the development of therapeutic agents blocking interaction signals between SPP1+ TAMs and cancer cells or targeting SPP1+ TAMs in cancer patients. Although the putative interaction analysis and correlation analysis between ligand and receptor cannot define the accurate causality, this indicates a potential role for cell-to-cell interactions *in vivo*.

## Data Availability Statement

The original contributions presented in the study are included in the article/Supplementary Material, further inquiries can be directed to the corresponding author/s.

## Author Contributions

JW, ZC, and HD conceptualized the study and involved in writing—review and editing. ZC, JW, and MH were involved in the methodology. ZC, ZH, and DJ provided the software. MH, JW, and JL performed the experiment validation. ZC and JW conducted the formal analysis and performed the investigation. ZC curated the data. JW and ZC were involved in writing—original draft preparation. HD was the project administrator and acquired funding. All authors contributed to the article and approved the submitted version.

## Conflict of Interest

The authors declare that the research was conducted in the absence of any commercial or financial relationships that could be construed as a potential conflict of interest.

## Publisher’s Note

All claims expressed in this article are solely those of the authors and do not necessarily represent those of their affiliated organizations, or those of the publisher, the editors and the reviewers. Any product that may be evaluated in this article, or claim that may be made by its manufacturer, is not guaranteed or endorsed by the publisher.

## Footnotes

1
http://blueprint.lambrechtslab.org

2
https://portal.gdc.cancer.gov/

3
http://www.gsea-msigdb.org/gsea/msigdb/

4
https://cibersort.stanford.edu

## References

[B1] Azizi E. Carr A. J. Plitas G. Cornish A. E. Konopacki C. Prabhakaran S. (2018). Single-cell map of diverse immune phenotypes in the breast tumor microenvironment. *Cell* 174 1293–1308.e36. 10.1016/J.Cell.2018.05.060 29961579 PMC6348010

[B2] Bhandari V. Hoey C. Liu L. Y. Lalonde E. Ray J. Livingstone J. (2019). Molecular landmarks of tumor hypoxia across cancer types. *Nat. Genet.* 51 308–318. 10.1038/S41588-018-0318-2 30643250

[B3] Boström P. Magnusson B. Svensson P. A. Wiklund O. Borén J. Carlsson L. M. (2006). Hypoxia converts human macrophages into triglyceride-loaded foam cells. *Arterioscler. Thromb. Vasc. Biol.* 26 1871–1876. 10.1161/01.Atv.0000229665.78997.0b 16741148

[B4] Chen P. Zhao D. Li J. Liang X. Li J. Chang A. (2019). Symbiotic macrophage-glioma cell interactions reveal synthetic lethality in pten-null glioma. *Cancer Cell* 35 868–884.e6. 10.1016/J.Ccell.2019.05.003 31185211 PMC6561349

[B5] Chen X. Song E. (2019). Turning foes to friends: targeting cancer-associated fibroblasts. *Nat. Rev. Drug Discov.* 18 99–115. 10.1038/S41573-018-0004-1 30470818

[B6] Cheng S. Li Z. Gao R. Xing B. Gao Y. Yang Y. (2021). A pan-cancer single-cell transcriptional atlas of tumor infiltrating myeloid cells. *Cell* 184 792–809.e23. 10.1016/J.Cell.2021.01.010 33545035

[B7] Cho W. Y. Hong S. H. Singh B. Islam M. A. Lee S. Lee A. Y. (2015). Suppression of tumor growth in lung cancer xenograft model mice by poly(Sorbitol-Co-Pei)-mediated delivery of osteopontin sirna. *Eur. J. Pharm. Biopharm.* 94 450–462. 10.1016/J.Ejpb.2015.06.017 26141346

[B8] Colegio O. R. Chu N. Q. Szabo A. L. Chu T. Rhebergen A. M. Jairam V. (2014). Functional polarization of tumour-associated macrophages by tumour-derived lactic acid. *Nature* 513 559–563. 10.1038/Nature13490 25043024 PMC4301845

[B9] Costa A. Kieffer Y. Scholer-Dahirel A. Pelon F. Bourachot B. Cardon M. (2018). Fibroblast heterogeneity and immunosuppressive environment in human breast cancer. *Cancer Cell* 33 463–479.e10. 10.1016/J.Ccell.2018.01.011 29455927

[B10] Efremova M. Vento-Tormo M. Teichmann S. A. Vento-Tormo R. (2020). Cellphonedb: inferring cell-cell communication from combined expression of multi-subunit ligand-receptor complexes. *Nat. Protoc.* 15 1484–1506. 10.1038/S41596-020-0292-X 32103204

[B11] Grada A. Otero-Vinas M. Prieto-Castrillo F. Obagi Z. Falanga V. (2017). Research techniques made simple: analysis of collective cell migration using the wound healing assay. *J. Invest. Dermatol.* 137 e11–e16. 10.1016/J.Jid.2016.11.020 28110712

[B12] Guo X. Zhang Y. Zheng L. Zheng C. Song J. Zhang Q. (2018). Global characterization of T cells in non-small-cell lung cancer by single-cell sequencing. *Nat. Med.* 24 978–985. 10.1038/S41591-018-0045-3 29942094

[B13] Haider S. Mcintyre A. Van Stiphout R. G. Winchester L. M. Wigfield S. Harris A. L. (2016). Genomic alterations underlie a pan-cancer metabolic shift associated with tumour hypoxia. *Genome Biol.* 17:140. 10.1186/S13059-016-0999-8 27358048 PMC4926297

[B14] Hänzelmann S. Castelo R. Guinney J. (2013). Gsva: gene set variation analysis for microarray and RNA-Seq data. *BMC Bioinformatics* 14:7. 10.1186/1471-2105-14-7 23323831 PMC3618321

[B15] Henze A. T. Mazzone M. (2016). The impact of hypoxia on tumor-associated macrophages. *J. Clin. Invest.* 126 3672–3679. 10.1172/Jci84427 27482883 PMC5096805

[B16] Hompland T. Fjeldbo C. S. Lyng H. (2021). Tumor hypoxia as a barrier in cancer therapy: why levels matter. *Cancers (Basel)* 13:499. 10.3390/Cancers13030499 33525508 PMC7866096

[B17] Ji A. L. Rubin A. J. Thrane K. Jiang S. Reynolds D. L. Meyers R. M. (2020). Multimodal analysis of composition and spatial architecture in human squamous cell carcinoma. *Cell* 182 1661–1662. 10.1016/J.Cell.2020.08.043 32946785 PMC7505493

[B18] Kale S. Raja R. Thorat D. Soundararajan G. Patil T. V. Kundu G. C. (2015). Osteopontin signaling upregulates cyclooxygenase-2 expression in tumor-associated macrophages leading to enhanced angiogenesis and melanoma growth via A 9β1 integrin. *Oncogene* 34 5408–5410. 10.1038/Onc.2015.315 26473949

[B19] Kieffer Y. Hocine H. R. Gentric G. Pelon F. Bernard C. Bourachot B. (2020). Single-cell analysis reveals fibroblast clusters linked to immunotherapy resistance in cancer. *Cancer Discov.* 10 1330–1351. 10.1158/2159-8290.Cd-19-1384 32434947

[B20] Lappano R. Talia M. Cirillo F. Rigiracciolo D. C. Scordamaglia D. Guzzi R. (2020). The Il1β-Il1r signaling is involved in the stimulatory effects triggered by hypoxia in breast cancer cells and Cancer-Associated Fibroblasts (Cafs). *J. Exp. Clin. Cancer Res.* 39:153. 10.1186/S13046-020-01667-Y 32778144 PMC7418191

[B21] Lee H. O. Hong Y. Etlioglu H. E. Cho Y. B. Pomella V. Van Den Bosch B. (2020). Lineage-dependent gene expression programs influence the immune landscape of colorectal cancer. *Nat. Genet.* 52 594–603. 10.1038/S41588-020-0636-Z 32451460

[B22] Leung D. W. Cachianes G. Kuang W. J. Goeddel D. V. Ferrara N. (1989). Vascular endothelial growth factor is a secreted angiogenic mitogen. *Science* 246 1306–1309. 10.1126/Science.2479986 2479986

[B23] Lu D. Y. Yeh W. L. Huang S. M. Tang C. H. Lin H. Y. Chou S. J. (2012). Osteopontin increases heme oxygenase-1 expression and subsequently induces cell migration and invasion in glioma cells. *Neuro. Oncol.* 14 1367–1378. 10.1093/Neuonc/Nos262 23074199 PMC3480271

[B24] Ma X. Bi E. Lu Y. Su P. Huang C. Liu L. (2019). Cholesterol induces Cd8+ T cell exhaustion in the tumor microenvironment. *Cell Metab.* 30 143–156.e5. 10.1016/J.Cmet.2019.04.002 31031094 PMC7061417

[B25] Mi Z. Bhattacharya S. D. Kim V. M. Guo H. Talbot L. J. Kuo P. C. (2011). Osteopontin promotes CCL5-mesenchymal stromal cell-mediated breast cancer metastasis. *Carcinogenesis* 32 477–487. 10.1093/Carcin/Bgr009 21252118 PMC3105582

[B26] Mi Z. Guo H. Russell M. B. Liu Y. Sullenger B. A. Kuo P. C. (2009). RNA aptamer blockade of osteopontin inhibits growth and metastasis of MDA-MB231 breast cancer cells. *Mol. Ther.* 17 153–161. 10.1038/Mt.2008.235 18985031 PMC2834992

[B27] Neumann C. Garreis F. Paulsen F. Hammer C. M. Birke M. T. Scholz M. (2014). Osteopontin is induced by TGF-B 2 and regulates metabolic cell activity in cultured human optic nerve head astrocytes. *PLoS One* 9:e92762. 10.1371/Journal.Pone.0092762 24718314 PMC3981660

[B28] Pang X. Xie R. Zhang Z. Liu Q. Wu S. Cui Y. (2019). Identification Of SPP1 as an extracellular matrix signature for metastatic castration-resistant prostate cancer. *Front. Oncol.* 9:924. 10.3389/Fonc.2019.00924 31620371 PMC6760472

[B29] Peng J. Sun B. F. Chen C. Y. Zhou J. Y. Chen Y. S. Chen H. (2019). Single-cell RNA-Seq highlights intra-tumoral heterogeneity and malignant progression in pancreatic ductal adenocarcinoma. *Cell Res.* 29 725–738. 10.1038/S41422-019-0195-Y 31273297 PMC6796938

[B30] Qian J. Olbrecht S. Boeckx B. Vos H. Laoui D. Etlioglu E. (2020). A pan-cancer blueprint of the heterogeneous tumor microenvironment revealed by single-cell profiling. *Cell Res.* 30 745–762. 10.1038/S41422-020-0355-0 32561858 PMC7608385

[B31] Rao G. Wang H. Li B. Huang L. Xue D. Wang X. (2013). Reciprocal interactions between tumor-associated macrophages and CD44-positive cancer cells via osteopontin/CD44 promote tumorigenicity in colorectal cancer. *Clin. Cancer Res.* 19 785–797. 10.1158/1078-0432.Ccr-12-2788 23251004

[B32] Robinson M. D. Mccarthy D. J. Smyth G. K. (2010). Edger: a bioconductor package for differential expression analysis of digital gene expression data. *Bioinformatics* 26 139–140. 10.1093/Bioinformatics/Btp616 19910308 PMC2796818

[B33] Scharping N. E. Rivadeneira D. B. Menk A. V. Vignali P. Ford B. R. Rittenhouse N. L. (2021). Mitochondrial stress induced by continuous stimulation under hypoxia rapidly drives T cell exhaustion. *Nat. Immunol.* 22 205–215. 10.1038/S41590-020-00834-9 33398183 PMC7971090

[B34] Thorsson V. Gibbs D. L. Brown S. D. Wolf D. Bortone D. S. Ou Yang T. H. (2019). The immune landscape of cancer. *Immunity* 51 411–412. 10.1016/J.Immuni.2019.08.004 31433971

[B35] Trapnell C. Cacchiarelli D. Grimsby J. Pokharel P. Li S. Morse M. (2014). The dynamics and regulators of cell fate decisions are revealed by pseudotemporal ordering of single cells. *Nat. Biotechnol.* 32 381–386. 10.1038/Nbt.2859 24658644 PMC4122333

[B36] Wai P. Y. Kuo P. C. (2004). The role of osteopontin in tumor metastasis. *J. Surg. Res.* 121 228–241. 10.1016/J.Jss.2004.03.028 15501463

[B37] Wang L. Li Y. S. Yu L. G. Zhang X. K. Zhao L. Gong F. L. (2020). Galectin-3 expression and secretion by tumor-associated macrophages in hypoxia promotes breast cancer progression. *Biochem. Pharmacol.* 178:114113. 10.1016/J.Bcp.2020.114113 32579956

[B38] Wei J. Huang K. Chen Z. Hu M. Bai Y. Lin S. (2020). Characterization of glycolysis-associated molecules in the tumor microenvironment revealed by pan-cancer tissues and lung cancer single cell data. *Cancers (Basel)* 12:1788. 10.3390/Cancers12071788 32635458 PMC7408567

[B39] Wei R. Wong J. Kwok H. F. (2017). Osteopontin – a promising biomarker for cancer therapy. *J. Cancer* 8 2173–2183. 10.7150/Jca.20480 28819419 PMC5560134

[B40] Xiao Z. Dai Z. Locasale J. W. (2019). Metabolic landscape of the tumor microenvironment at single cell resolution. *Nat. Commun.* 10:3763. 10.1038/S41467-019-11738-0 31434891 PMC6704063

[B41] Yona S. Kim K. W. Wolf Y. Mildner A. Varol D. Breker M. (2013). Fate mapping reveals origins and dynamics of monocytes and tissue macrophages under homeostasis. *Immunity* 38 79–91. 10.1016/J.Immuni.2012.12.001 23273845 PMC3908543

[B42] Zhou Y. Zhou B. Pache L. Chang M. Khodabakhshi A. H. Tanaseichuk O. (2019). Metascape provides a biologist-oriented resource for the analysis of systems-level datasets. *Nat. Commun.* 10:1523. 10.1038/S41467-019-09234-6 30944313 PMC6447622

