# Supplementary figures and images for: Characterizing Intercellular Communication of Pan-Cancer Reveals SPP1+ Tumor-Associated Macrophage Expanded in Hypoxia and Promoting Cancer Malignancy Through Single-Cell RNA-Seq Data
Source: Front Cell Dev Biol. 2021 Oct 5;9:749210. doi: 10.3389/fcell.2021.749210 (PMC8523849; doi:10.3389/fcell.2021.749210)

A

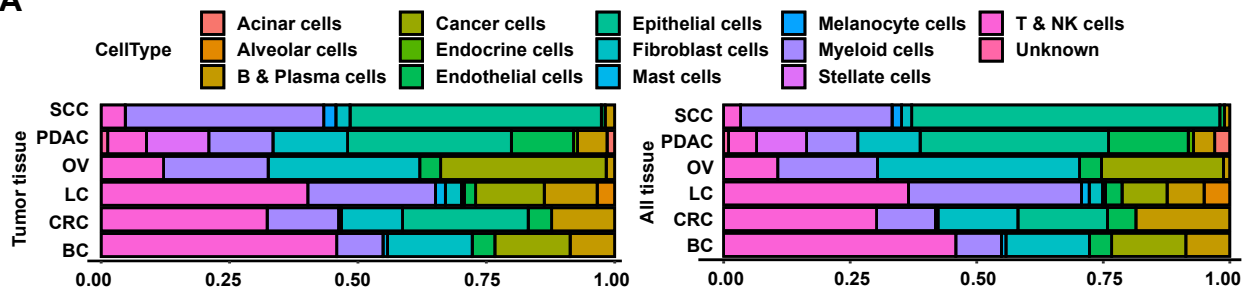

B

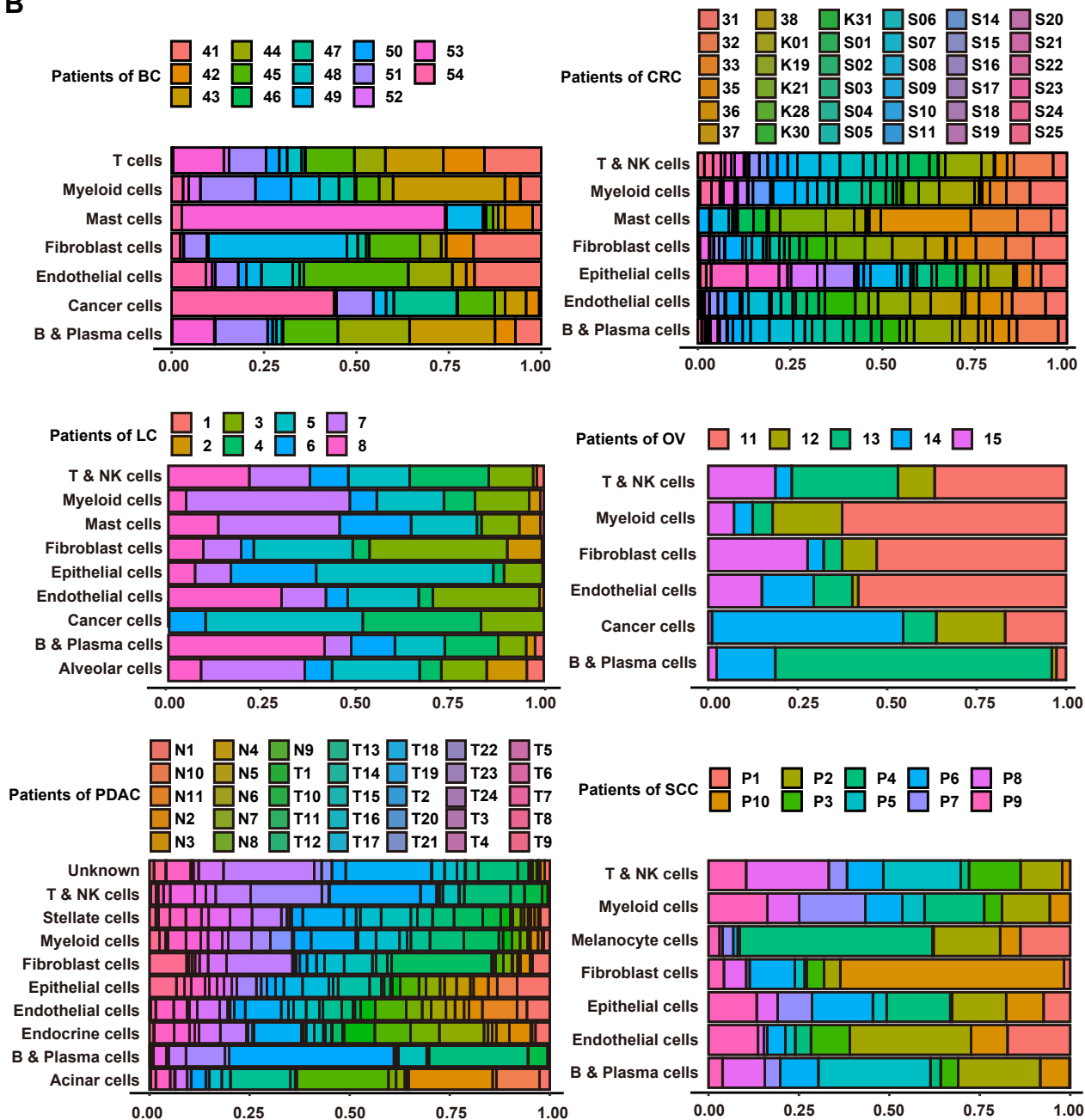

Supplement: Supplementary file 9 [file Data_Sheet_1.PDF]

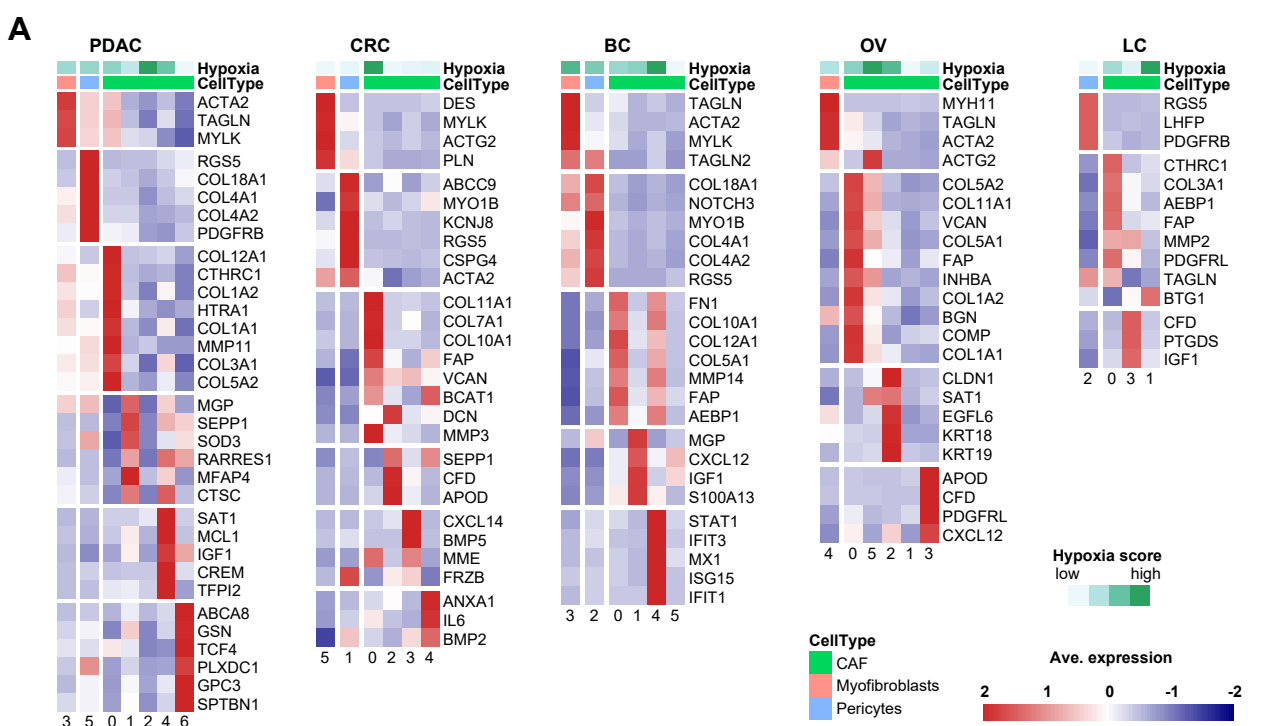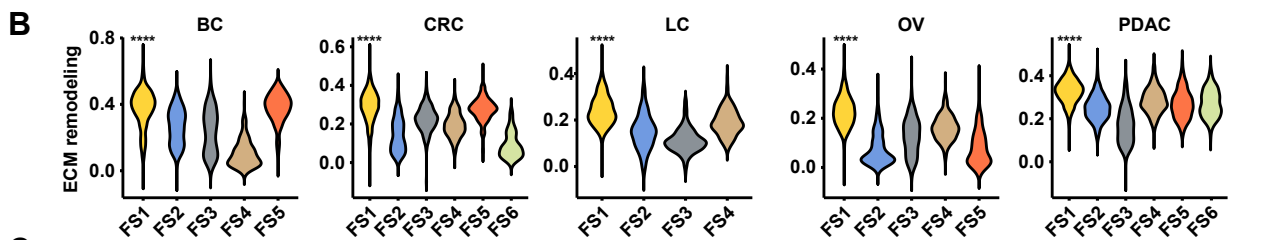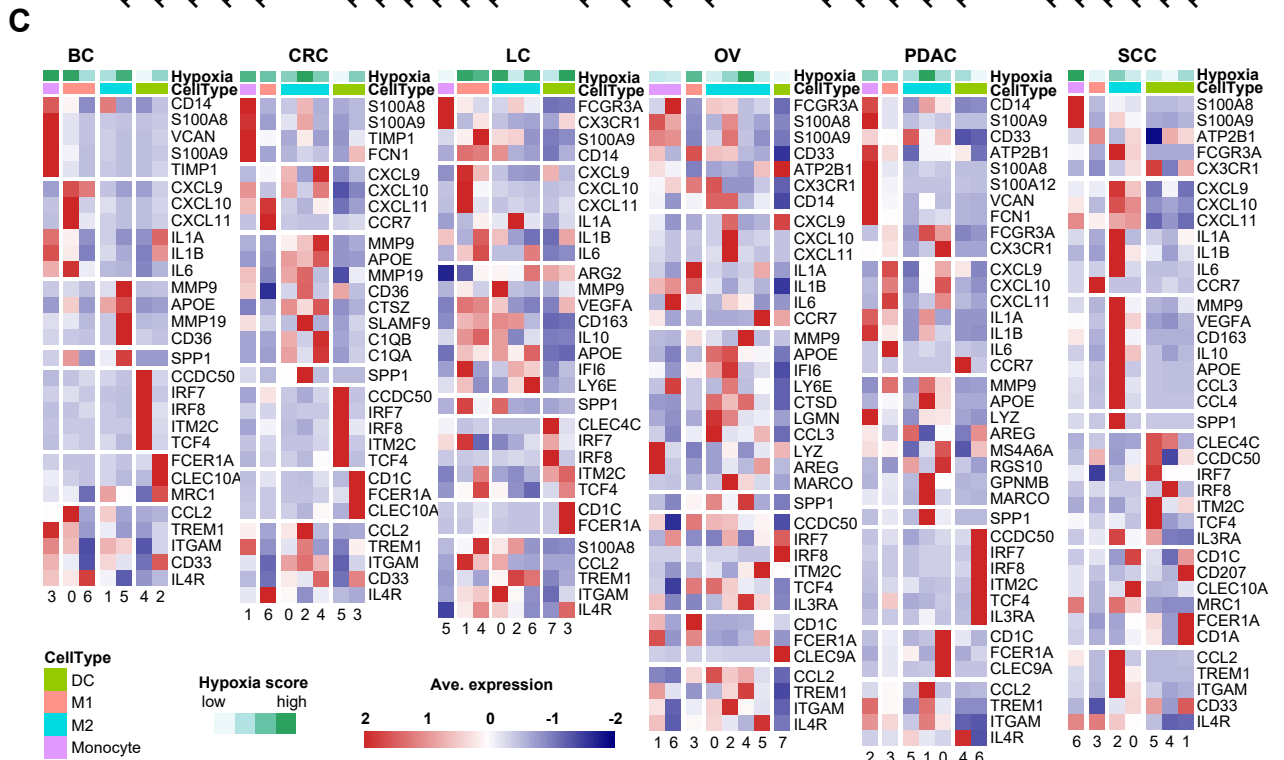

Supplement: Supplementary file 10 [file Data_Sheet_2.PDF]

**A**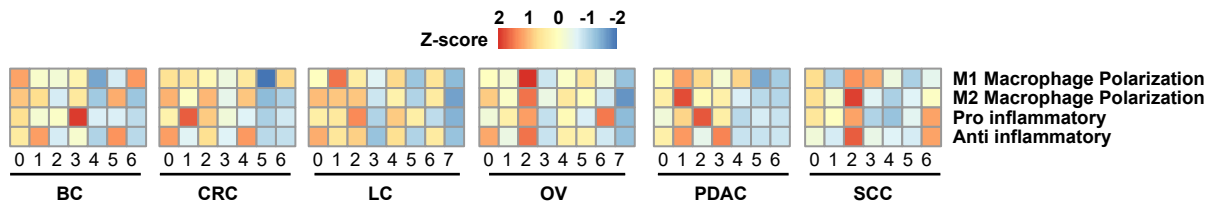**B**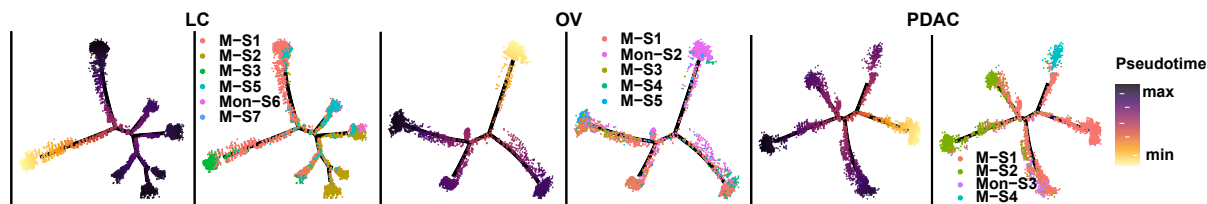**C**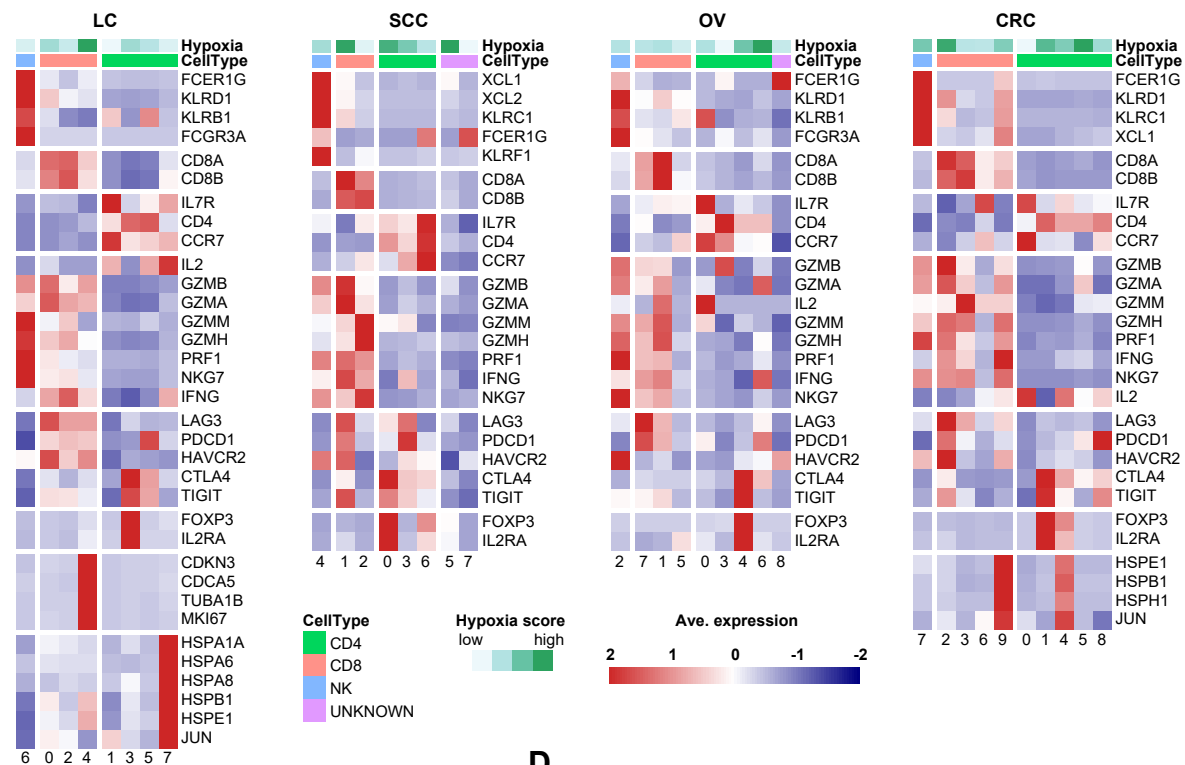**D**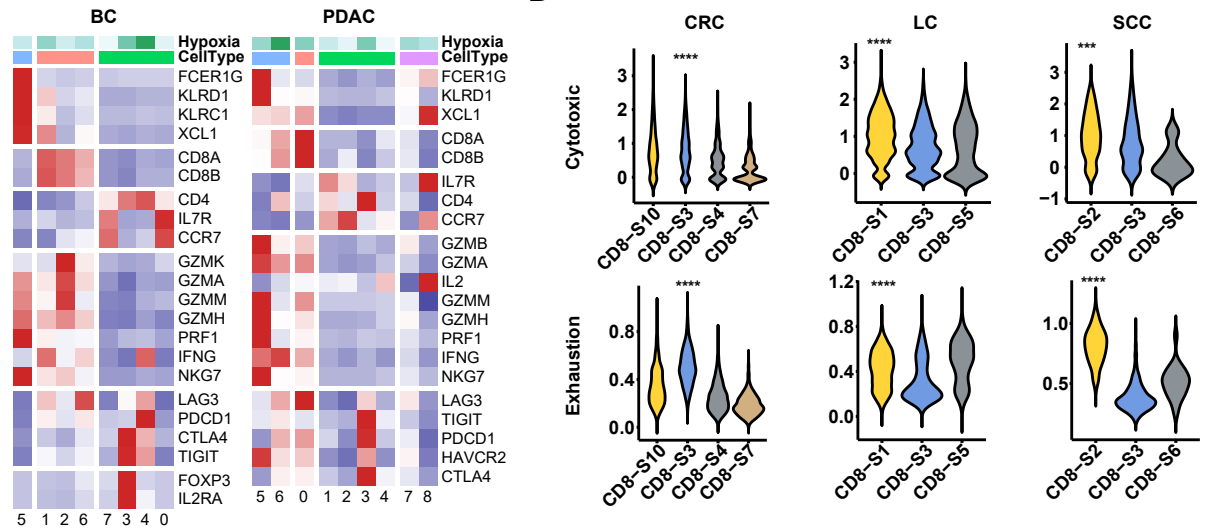

Supplement: Supplementary file 11 [file Data_Sheet_3.PDF]

**A**

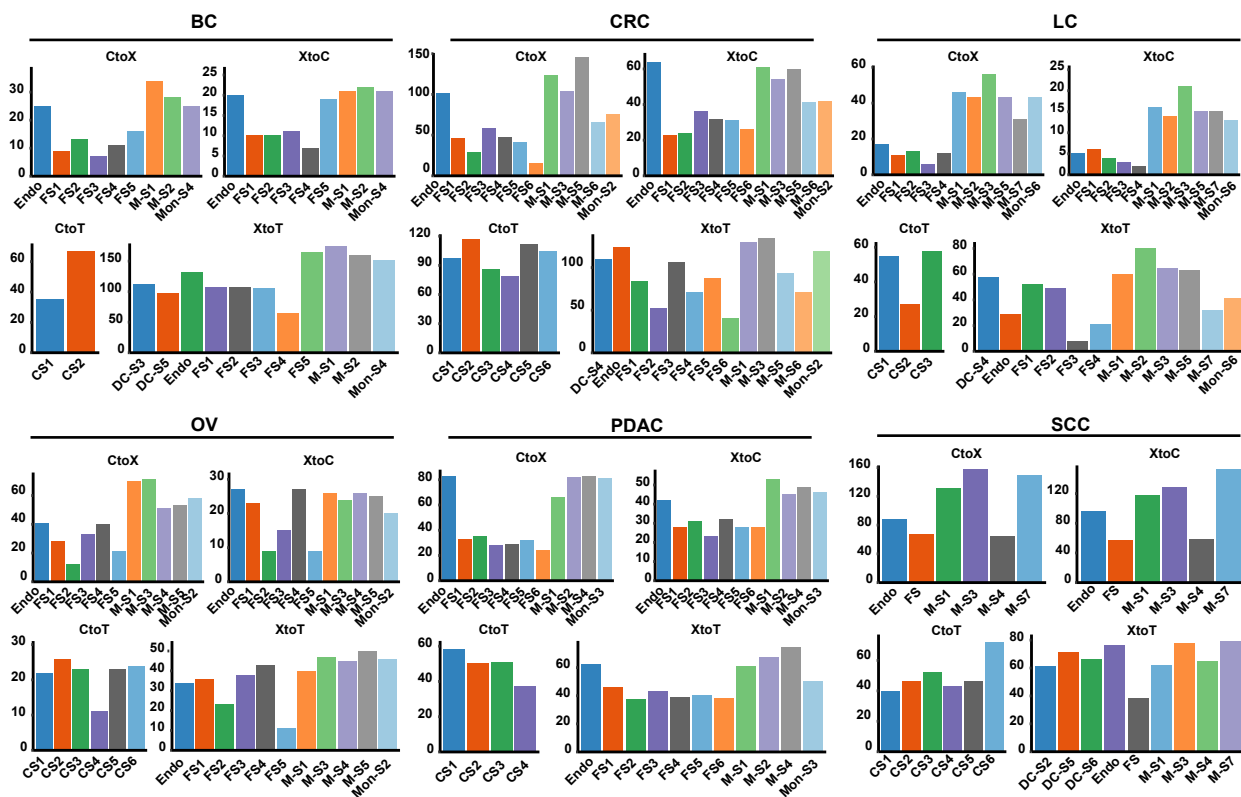

# B

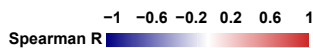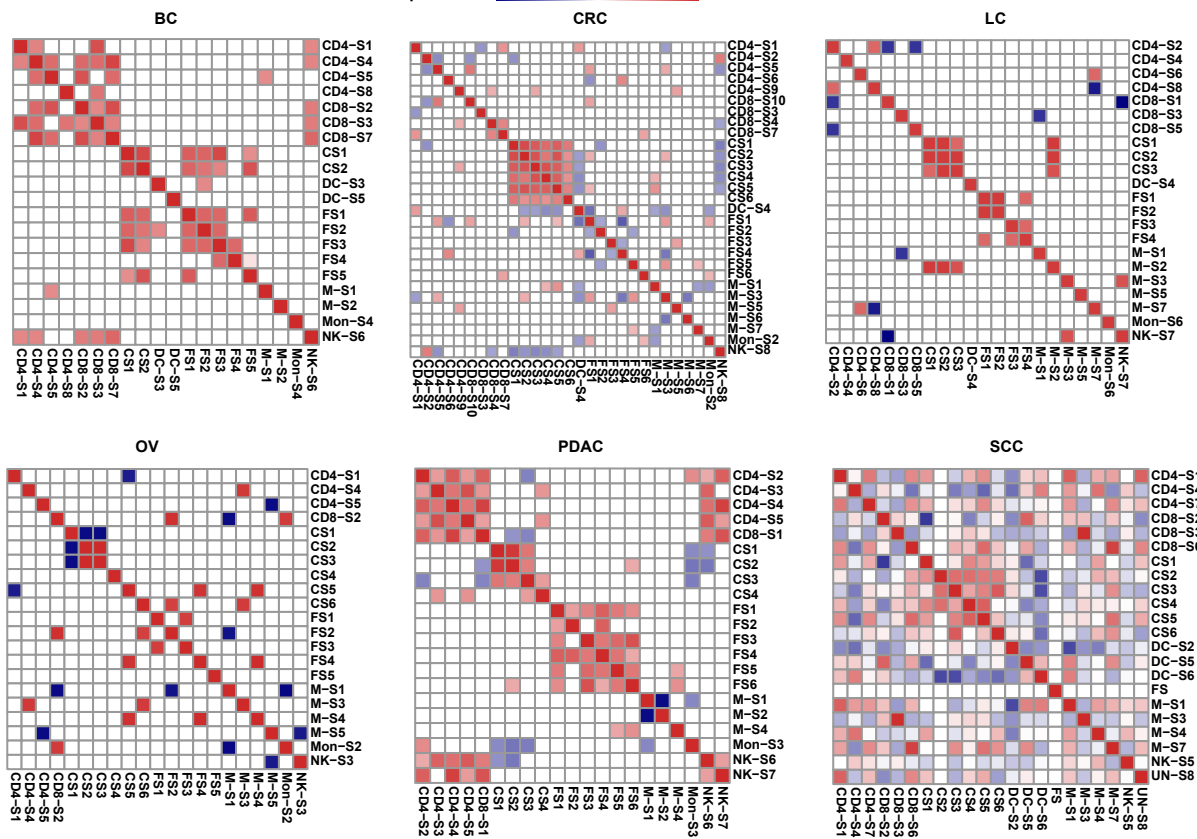

Supplement: Supplementary file 12 [file Data_Sheet_4.PDF]

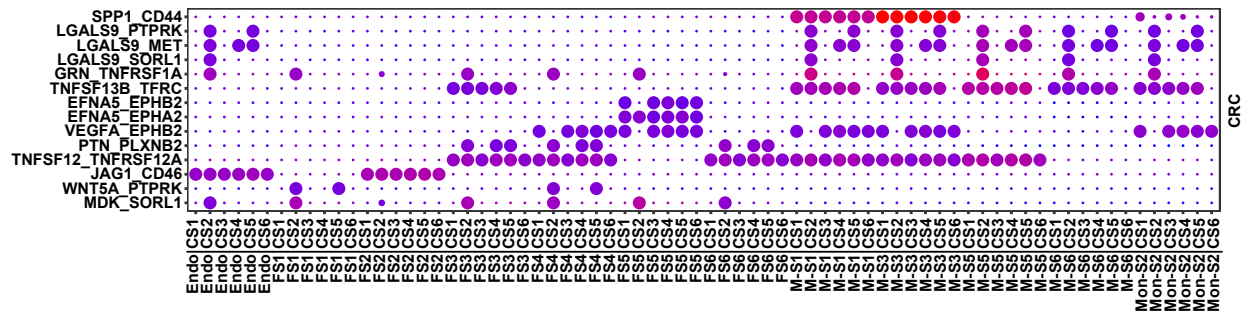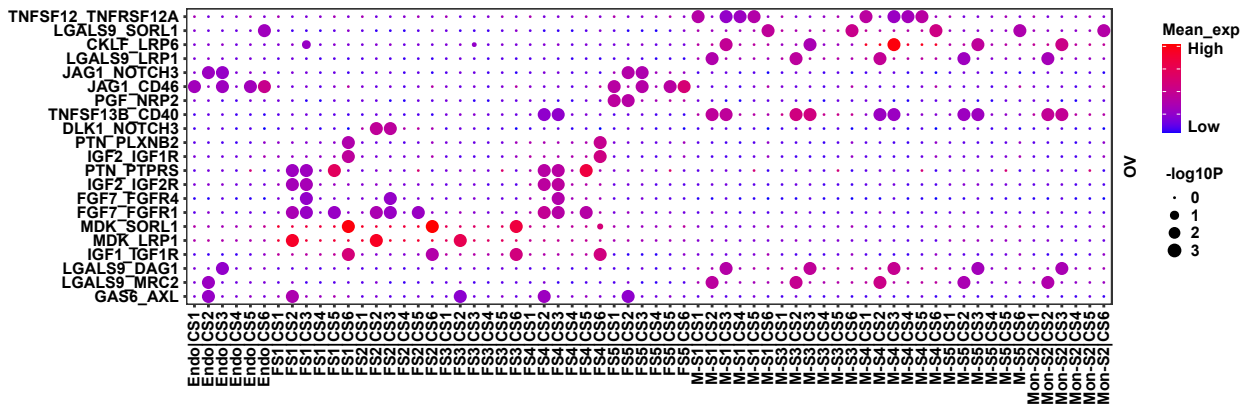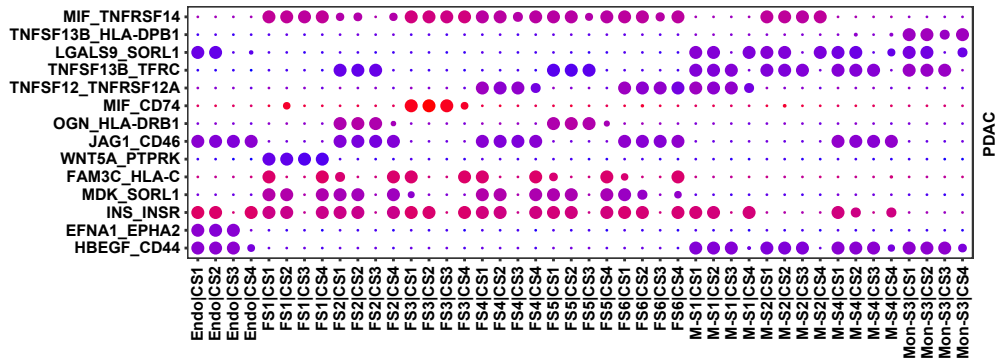

Supplement: Supplementary file 13 [file Data_Sheet_5.PDF]

**A**

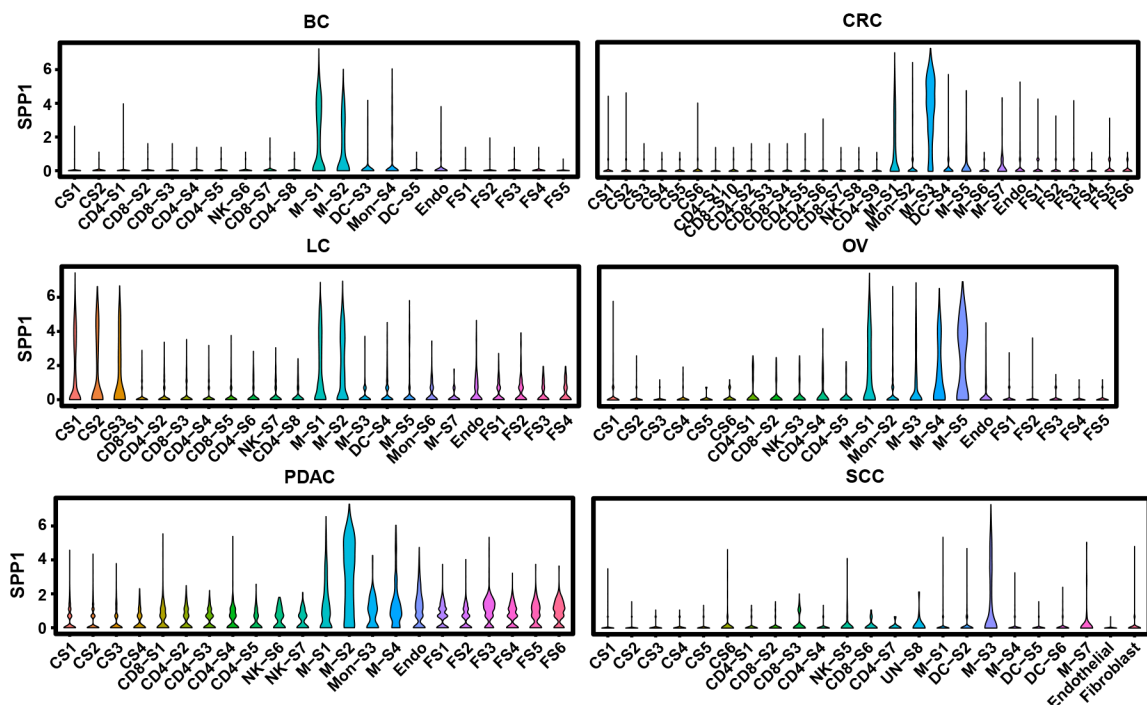

**B**

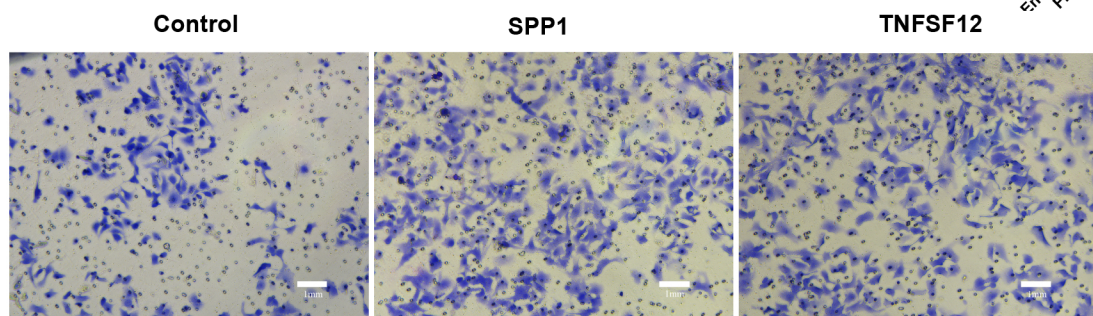

**C**

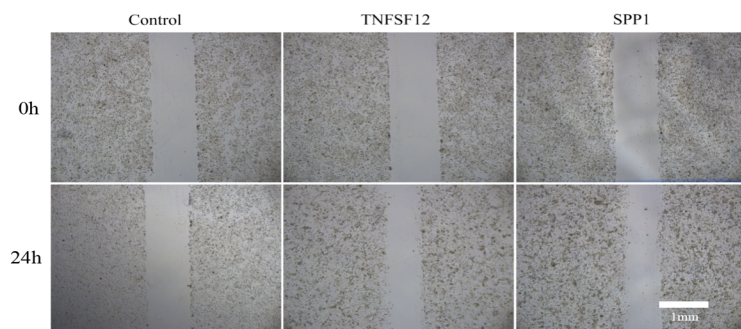

**D**

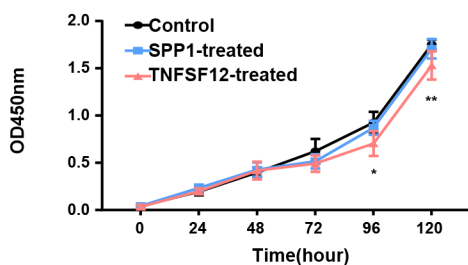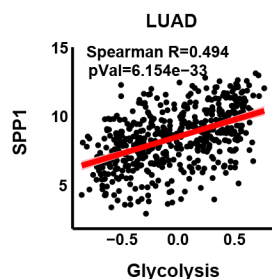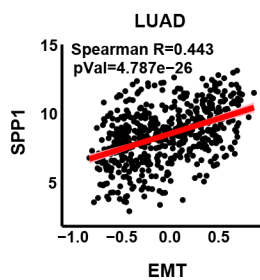

**E**

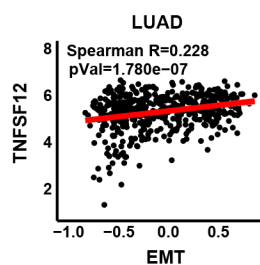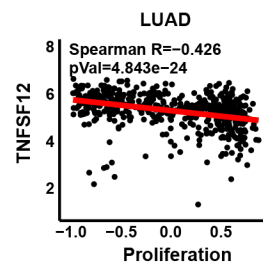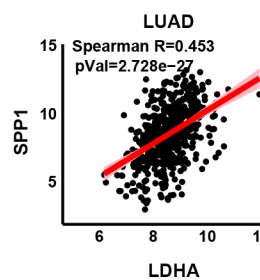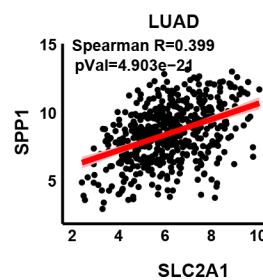

Supplement: Supplementary file 14 [file Data_Sheet_6.PDF]

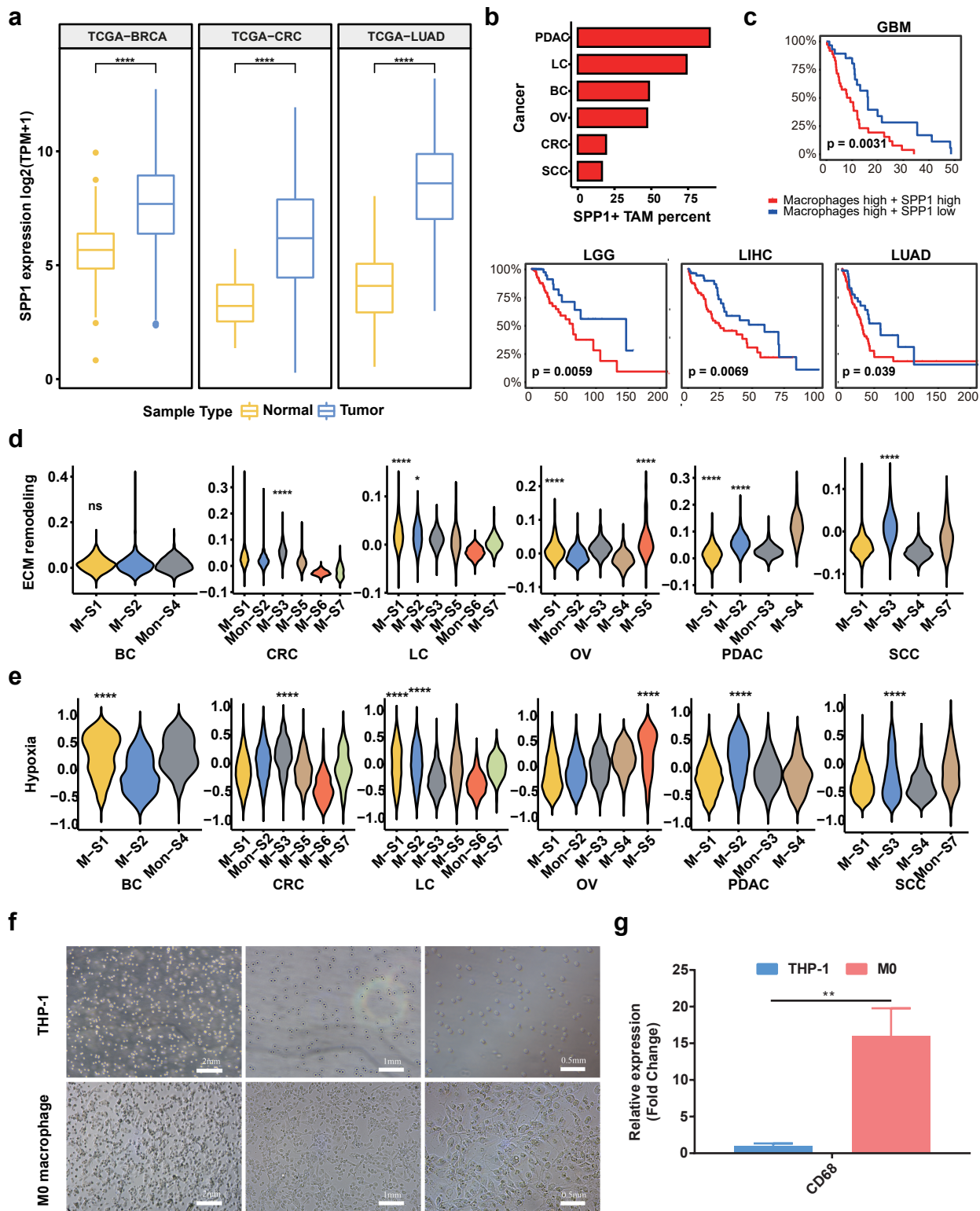

Supplement: Supplementary file 15 [file Data_Sheet_7.PDF]

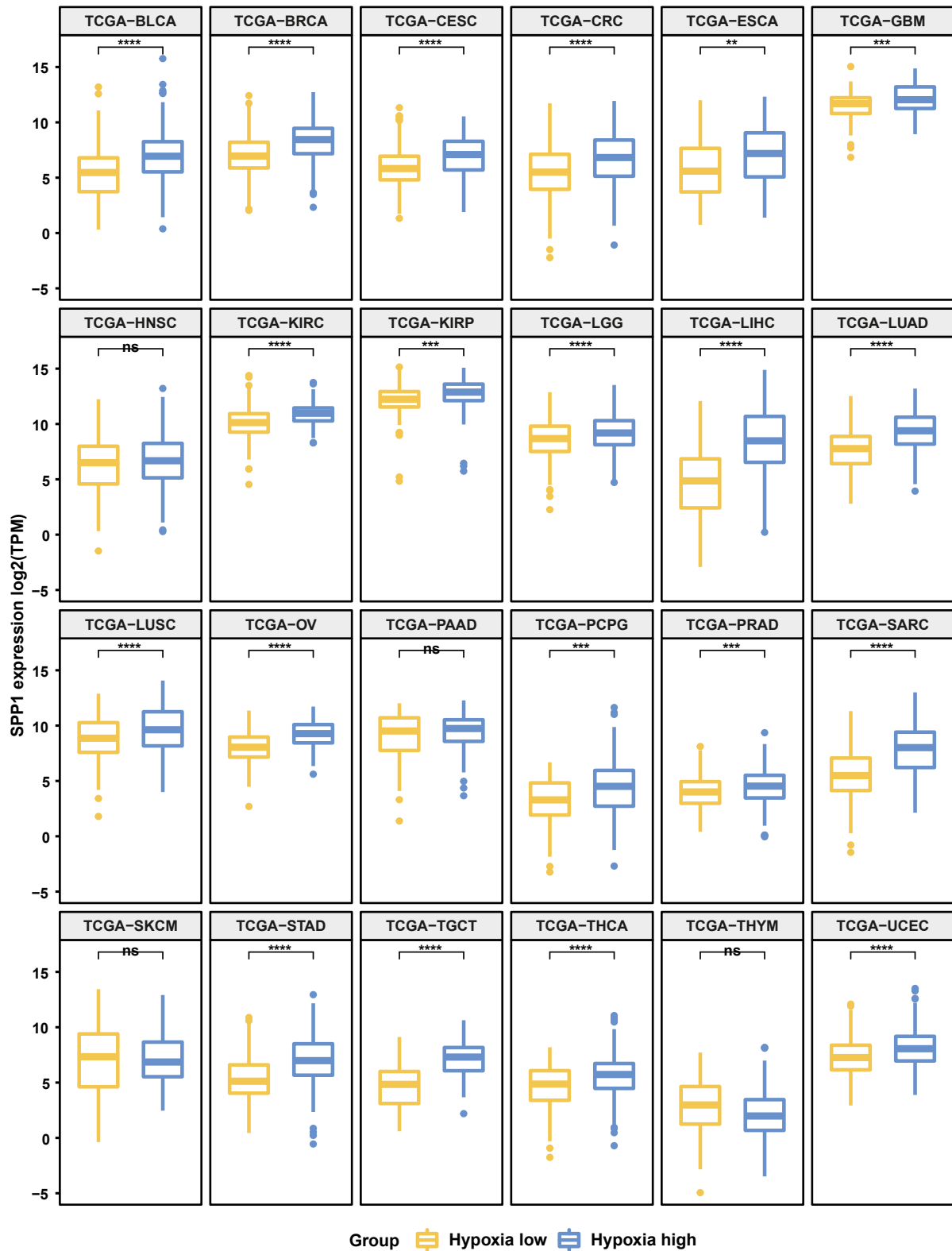

Supplement: Supplementary file 16 [file Data_Sheet_8.PDF]

A

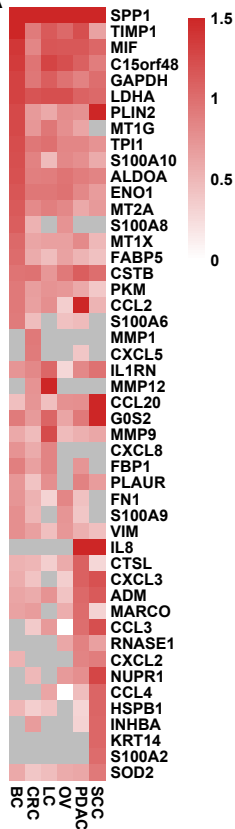

B

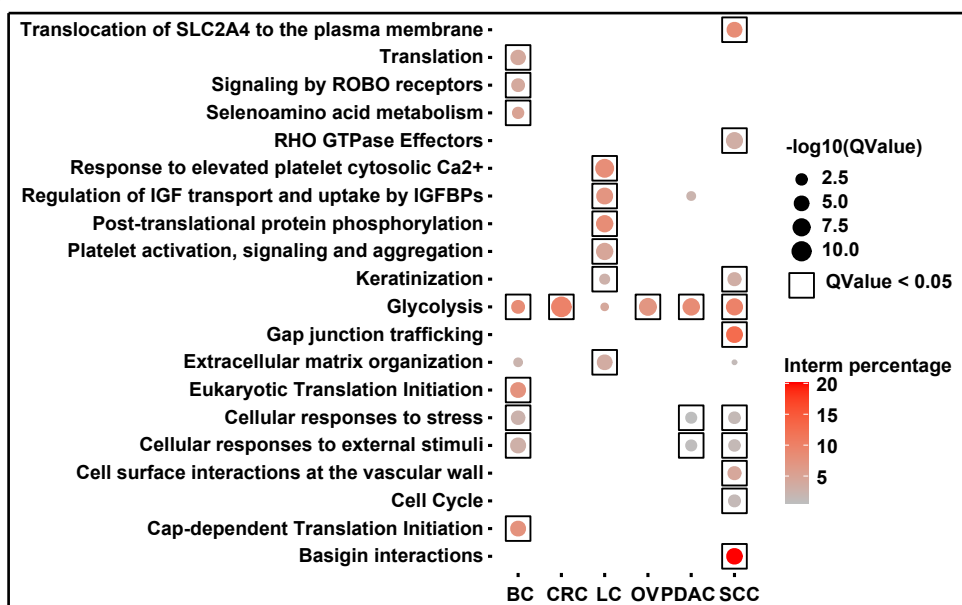

Supplement: Supplementary file 17 [file Data_Sheet_9.PDF]
