# Supplementary material for: Characterizing Intercellular Communication of Pan-Cancer Reveals SPP1+ Tumor-Associated Macrophage Expanded in Hypoxia and Promoting Cancer Malignancy Through Single-Cell RNA-Seq Data
Source: Front Cell Dev Biol. 2021 Oct 5;9:749210. doi: 10.3389/fcell.2021.749210 (PMC8523849; doi:10.3389/fcell.2021.749210)
Supplement: Supplementary file 18 [file Data_Sheet_10.DOCX]

Supplementary Material

# Supplementary Tables

Table S1. Sample size and cell number of single cells across six cancer types.

Table S2. The definition of sub-cluster and the marker gene list for cluster and subcluster for each cell types.

Table S3. Gene list of each gene signatures analyzed in this study.

Table S4. Primer sequence used in this study.

Table S5. The ligand receptor pairs among different cell types.

Table S6. The proportion of each cell subcluster across patient in six cancer types.

Table S7. The correlation coefficient between receptors with specific genes and gene sets in cancer cells and the SPP1 expression in  single macrophage across 6 cancer types.

Table S8. The different expressed genes in hypoxia single cells and hypoxia TCGA cancer.

# Supplementary Figures

Figure S1. Cell type identification in pan-cancer landscape. (A). The proportion of cells that contributed to tumor tissues (left) and all tissues (right), colored by cell type. (B). The proportion of cells that contributed to each cell type by each sample, colored by sample.

Figure S2. Subtype classification and characteristics of stroma cells and myeloid cells across six cancer types. (A). Heatmap of Z-score normalized log2 (count+1) expression of canonical marker genes for fibroblasts. The color of square on the top map indicates the average hypoxia score for each fibroblast clusters (light green to green). The expression was centered to the average log2 (count+1) expression across all single cells with a scale from 2 to −2. (B). Hallmark gene set scores for extracellular matrix (ECM) remodeling for all stroma cell clusters in BC, CRC, LC, OV, and PDAC. FS1, FS2, FS3, FS4, and FS5 are the subcluster 0, 1, 2, 3, and 4 of stroma cells. ****p≤0.0001, two-sided Wilcoxon rank sum test. (C). Heatmap of marker genes for myeloid cells (see A).

Figure S3. Subtype classification and characteristics of myeloid cells, T and NK cells across cancer types. (A). Heatmap of specific gene set calculated by GSVA score for myeloid cells across six cancer types. (B). The branched trajectory of myeloid cells state transition in LC, OV and PDAC inferred by Monocle 2. Each dot corresponds to one single cell, colored according to its cluster label. Subclusters 0, 1, 2, 3, 4 of macrophages/monocytes were labeled M-S1/Mon-S1, M-S2/Mon-S2, M-S3/Mon-S3, M-S4/Mon-S4, M-S5/Mon-S5 in each cancer types, see Figure 2. (C). Heatmap of Z-score normalized log2 (count+1) expression of canonical marker genes for T and NK cells. The color of square on the top map indicates the average hypoxia score for each T and NK cell clusters (light green to green). The expression was centered to the average log2 (count+1) expression across all single cells with a scale from 2 to −2. (D). The cytotoxicity (top) and exhaustion (bottom) score for CD8 T cell clusters in BC, CRC, LC and SCC, respectively. *** p≤0.001, ****p≤0.0001, two-sided Wilcoxon rank sum test.

Figure S4. The significant ligand receptor pairs numbers and correlation matrix between different cell types. (A). The number of significant ligand-receptor pairs between cell groups predicted by CellphoneDB. (B). Correlation matrix between proportion of cell subpopulations across all cell types. The proportion of each cell subcluster was estimated with respect to all cells of same cell type in each sample. Subclusters 0, 1, 2, 3, 4 of stroma cells were labeled FS1, FS2, FS3, FS4, FS5 in each cancer types. Subclusters 0, 1, 2, 3, 4 of cancer cells were labeled CS1, CS2, CS3, CS4, CS5 in each cancer types. Subclusters 0, 1, 2, 3, 4 of macrophages/monocytes/ dendritic cells were labeled M-S1/Mon-S1/DC-S1, M-S2/Mon-S2/DC-S2, M-S3/Mon-S3/DC-S3, M-S4/Mon-S4/DC-S4, M-S5/Mon-S5/DC-S5 in each cancer types. Endo was endothelial cells.

Figure S5. Significant ligand-receptor pair genes accounting for specific inter-cellular interactions in CRC, OV and PDAC, respectively. P values indicated by circle size. The means of the average expression level of interacting molecule 1 in cluster 1 and interacting molecule 2 in cluster 2 are indicated by color, see Figure 4A.

Figure S6. TAMs promote glycolysis, angiogenesis and EMT of cancer cells. (A). Violin plot shows the expression of SPP1 in all cell subclusters in BC, CRC, LC, OV, PDAC, and SCC, respectively. Subclusters 0, 1, 2, 3, 4 of stroma cells were labeled FS1, FS2, FS3, FS4, FS5 in each cancer types. Subclusters 0, 1, 2, 3, 4 of cancer cells were labeled CS1, CS2, CS3, CS4, CS5 in each cancer types. Subclusters 0, 1, 2, 3, 4 of macro-phages/monocytes/ dendritic cells were labeled M-S1/Mon-S1/DC-S1, M-S2/Mon-S2/DC-S2, M-S3/Mon-S3/DC-S3, M-S4/Mon-S4/DC-S4, M-S5/Mon-S5/DC-S5 in each cancer types. Endo was endothelial cells. (B). Invasion cells in transwell assays in per field were compared between recombinant protein SPP1 or TNFSF12 added group and control group. (C). Cell migration area were compared between recombinant protein SPP1 or TNFSF12 added group and control group (D). Cell viability in A549 cancer cells exposed to TNFSF12 or SPP1 in day 1, 2, 3, 4, 5 and 6, respectively. *p≤0.05, **p≤0.01, two-sided unpaired t test. (E). Correlation scatter plot between TNFSF12 or SPP1 expression and specific genes or signatures in TCGA LUAD.

Figure S7. Characteristics of SPP1+TAMs in various cancer types. (A). Boxplot showing the com-parison of SPP1 expression in cancer and normal samples. ****p≤0.0001, two-sided Wilcoxon rank sum test. (B). The proportion of SPP1+TAMs across all myeloid cell sub-populations. (C). The Kaplan-Meier overall survival curves of TCGA patients grouped by the gene expression of SPP1 combined with macrophage abundance. (D). Violin plot shows the ECM remodeling score is higher in SPP1+TAMs subclusters across five cancer types. *p≤0.05, ****p≤0.0001, two-sided Wilcoxon rank sum test. Subclusters 0, 1, 2, 3, 4 of macrophages/monocytes/ dendritic cells were labeled M-S1/Mon-S1/DC-S1, M-S2/Mon-S2/DC-S2, M-S3/Mon-S3/DC-S3, M-S4/Mon-S4/DC-S4, M-S5/Mon-S5/DC-S5 in each cancer types. (E). Violin plot shows the hypoxia score is higher in SPP1+TAMs subclusters in across six cancer types. ****p≤0.0001, two-sided Wilcoxon rank sum test. (F). Macrophages is differentiated from THP-1 monocyte. (G). The expression of macrophages marker CD68 is up-regulated in THP-1- derived macrophages compared with THP-1 cells. **p≤0.01, two-sided unpaired t test.

Figure S8. Boxplot showing the comparison of SPP1 expression in hypoxia-high and -low samples across pan-cancer. **p≤0.01, ***p≤0.001, ****p≤0.0001, two-sided Wilcoxon rank sum test.

Figure S9. Molecular characteristics of different cell types under the hypoxia TME. (A). Heatmap depicts the DEGs between myeloid cells with high and low hypoxia score. (B). Enriched pathways in hypoxia-high cancer cells.
